# Toxicogenomic Profiling of 28 Nanomaterials in Mouse Airways

**DOI:** 10.1002/advs.202004588

**Published:** 2021-03-08

**Authors:** Pia A. S. Kinaret, Joseph Ndika, Marit Ilves, Henrik Wolff, Gerard Vales, Hannu Norppa, Kai Savolainen, Tiina Skoog, Juha Kere, Sergio Moya, Richard D. Handy, Piia Karisola, Bengt Fadeel, Dario Greco, Harri Alenius

**Affiliations:** ^1^ Institute of Biotechnology, Helsinki Institute of Life Science University of Helsinki Helsinki 00790 Finland; ^2^ Faculty of Medicine and Health Technology Tampere University Tampere 33720 Finland; ^3^ Human Microbiome Research Program (HUMI) University of Helsinki Helsinki 00014 Finland; ^4^ Finnish Institute of Occupational Health Helsinki 00250 Finland; ^5^ Department of Biosciences and Nutrition Karolinska Institutet Stockholm 141 83 Sweden; ^6^ Center for Cooperative Research in Biomaterials (CIC biomaGUNE) Basque Research and Technology Alliance (BRTA) Donostia‐San Sebastián 20014 Spain; ^7^ School of Biological & Marine Sciences University of Plymouth Plymouth PL4 8AA UK; ^8^ Institute of Environmental Medicine Karolinska Institutet Stockholm 171 77 Sweden; ^9^ BioMediTech Institute Tampere University Tampere 33520 Finland; ^10^ Finnish Center for Alternative Methods (FICAM) Tampere 33520 Finland

**Keywords:** airway exposure, immunotoxicity, nanomaterials, nanoparticles, nanotoxicology, toxicogenomics, transcriptomics

## Abstract

Toxicogenomics opens novel opportunities for hazard assessment by utilizing computational methods to map molecular events and biological processes. In this study, the transcriptomic and immunopathological changes associated with airway exposure to a total of 28 engineered nanomaterials (ENM) are investigated. The ENM are selected to have different core (Ag, Au, TiO_2_, CuO, nanodiamond, and multiwalled carbon nanotubes) and surface chemistries (COOH, NH_2_, or polyethylene glycosylation (PEG)). Additionally, ENM with variations in either size (Au) or shape (TiO_2_) are included. Mice are exposed to 10 µg of ENM by oropharyngeal aspiration for 4 consecutive days, followed by extensive histological/cytological analyses and transcriptomic characterization of lung tissue. The results demonstrate that transcriptomic alterations are correlated with the inflammatory cell infiltrate in the lungs. Surface modification has varying effects on the airways with amination rendering the strongest inflammatory response, while PEGylation suppresses toxicity. However, toxicological responses are also dependent on ENM core chemistry. In addition to ENM‐specific transcriptional changes, a subset of 50 shared differentially expressed genes is also highlighted that cluster these ENM according to their toxicity. This study provides the largest in vivo data set currently available and as such provides valuable information to be utilized in developing predictive models for ENM toxicity.

## Graphical abstract

C57BL/6 mice are exposed by oropharyngeal aspiration to 28 different engineered nanomaterials (ENM). Histological and cytological evaluation together with transcriptomics analyses reveals the important effect of surface functionalization (amination, carboxylation, or pegylation) as well as the significant role of the ENM core chemistry.

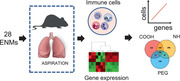

## 1 Introduction

In conventional toxicology, observing the histological changes, immune cell infiltration, phenotypic alterations, or behavioral characteristics after exposures to toxicants helps us to understand the underlying inflammatory responses. Nonetheless, these measures focus on specific endpoints, not revealing detailed molecular mechanisms underlying the toxic immune responses that possibly lead to long term consequences.^[^ ^1^ ^]^ Engineered nanomaterials (ENM) with complex features make classical toxicology approaches challenging as their toxicity is mediated by their physical and chemical properties; including, but not limited to size, shape, surface charge, aspect ratio, and functionalization. A shift from basic toxicity endpoint studies is needed in order to understand ENM mechanisms of toxicity and develop predictive computational models for ENM hazard assessment.^[^ ^1^, ^2^, ^3^ ^]^ For this, toxicogenomics, which seeks to identify relationships between changes in intracellular molecular profiles and exposure to suspected toxicants, provides a great opportunity for the toxicity assessment of ENM.

The problematic assessment of ENM toxicity is mostly due to complex interactions between nanoparticles and biosystems. The cellular uptake and toxic potential of ENM can be considerably altered by changing the surface chemistry of the core material. Different functional groups on the surface of ENM can alter the material properties through changes in the aggregation behavior, surface adsorption, or binding. For example, carboxylation (‐COOH) has been shown to reduce bioactivity and pathogenicity of multiwalled carbon nanotubes (MWCNT) and titanium nanoparticles.^[^ ^4^, ^5^ ^]^ On the other hand, Bonventre et al. reported that aminated (‐NR^3+^) Ag nanoparticles with silica shells and silica nanoparticles are more toxic than the same particles with hydroxyl‐terminated functionalization.^[^ ^6^ ^]^ Another surface modification that has been often tested is ENM functionalization with polyethylene glycosylation (‐PEG). PEG generates a biocompatible hydrophilic surface and has the ability to shield the core particle from immunosurveillance.^[^ ^7^ ^]^

In addition to functionalization associated toxicity, size and shape also direct the relative toxicity of ENM. For example, nano‐sized metal particles including copper oxide (CuO), gold (Au), and silver (Ag) are more toxic than larger particles with the same composition, probably due to enhanced uptake and dissolution when compared to larger particles.^[^ ^8^ ^]^ Due to their industrial utility, titanium dioxide nanoparticles (TiO_2_) are by far the most manufactured nanomaterials. Harmful effects that are dependent on particle shape and size have been documented in several in vitro and in vivo studies. Similar to CuO nanoparticles, the toxic potential of TiO_2_ increases as particle size decreases.^[^ ^9^ ^]^ CuO mechanisms of toxicity include excess reactive oxygen species (ROS) production and cell membrane damage.^[^ ^10^ ^]^ In addition, we, among others, have previously shown that carbon nanotubes elicit immune responses and certain MWCNT cause Th2‐type of inflammation, including strong eosinophilic influx to airways.^[^ ^11^, ^12^ ^]^

The toxicity and inflammatory potential of ENM are commonly investigated individually either in in vitro or in vivo settings. Systems biology and toxicogenomic methods enable identification of more specific exposure signatures that can be used for read‐across to facilitate toxicity screening of the vast array of industrial and medically relevant nanoparticles.^[^ ^13^ ^]^ However, comparison of the toxic effects of different ENM in these scattered studies is very difficult due to large differences in methodologies, models, and exposure protocols. Moreover, the number of studies leveraging omics methodologies to profile the mechanisms of action of more than a handful of ENM, especially in animal models, is limited. In the present study, we have performed by far the largest comparison of transcriptional and phenotypic effects, triggered by exposure to 28 exhaustively characterized ENM, with five different core chemistries and their functionalized counterparts including NH^3+^, COOH, and PEG functional groups. This large multiparametric, toxicological in vivo dataset provides important information about the cellular and molecular perturbations underlying ENM‐induced toxicity, thus facilitating hazard assessment and toxicity predictions.

## 2 Results and Discussion

### 2.1 Immune Cell Influx and Lung Histology Suggest Immunotoxic Potential of Some ENM

Overall study design is shown in Figure S1, Supporting Information. It includes comparison of phenotypic and transcriptional changes elicited by oropharyngeal aspiration exposure to 28 ENM (**Table** **1**) with five different core chemistries and their functionalizations including NH^3+^, COOH, and PEG functional groups. Based on our previous studies, a dose of 10 µg per day for four consecutive days elicits optimal inflammatory responses without more unspecific lung injury‐related responses from excessive material intake and possible agglomeration. Moreover, 4‐day exposure scenario ensures observation of explicit, ENM‐induced effects rather than more general acute effects, taking into account also the possible adaptive ^immunity^ activation.^[^ ^7^, ^12^ ^]^ Surface functionalization of the ENM was proven by X ray photoelectron spectroscopy (XPS), which allowed for detection of bands characteristic to the surface functionalization. Surface functionalization will impact on the charge of the ENM and this was characterized by zeta potential measurements. Notably, the zeta potentials of most of the materials were as expected, with a net negative charge on COOH‐functionalized particles, a lesser negative charge on the PEG, and positive charge of the amine functionalized materials, respectively. However, there were some exceptions with the MWCNT^NH2^ having a negative zeta potential and the TiO2^PEG^ rods with apparent positive charge (Table 1). The zeta potential measurement is based on the electrokinetic charge density of assumed spherical particles and is not intended for high aspect ratio materials such as MWCNT.^[^ ^14^ ^]^ The absolute values of zeta potential are also influenced by the ratio of bulk ions (i.e., NaCl) to particle numbers in the dispersion.^[^ ^15^ ^]^ Thus for MWCNT values should only be considered as a relative measure compared to the core. Nonetheless, the zeta potentials within the MWCNT are in exactly the same ranking as their aggregation behavior in NaCl solution.^[^ ^15^ ^]^ Similar arguments apply to the TiO_2_ rods. Zeta potential differences can be also partly explained by the nature of the ligands used for the different ENM. For CuO, the basic ligand for functionalization is a thiol with a carboxylate end group and PEG was attached forming an ester with the carboxylate. The negative charges for PEG CuO^PEG^ arise from possible residual COOH. For TiO_2_ the basic ligands for functionalization were a silane with an amine group and PEG was attached through an amide while the positive charges are due to non‐modified amines.

**Table 1:** Engineered nanomaterials and their physicochemical properties. *primary particle size was not measurable (below the resolution limits). Modified and reproduced with permission.^[^ ^35^ ^]^ Copyright 2020, Wiley

Particle	Estimated primary size L = µm, D = nm	Z‐potential (in water, [mV])
MWCNT^core^	L: 0.9–1.2/D: 10–15	−24 ± 1
MWCNT^NH2^	L: 0.6–0.9/D: 10–15	−21 ± 1
MWCNT^COOH^	L: 1.2–1.5/D: 10–15	−30 ± 2
MWCNT^PEG^	L: 0.6–0.9/D: 10–15	−2 ± 1
CuO^core^	D: 10–20	14.0 ± 1.2
CuO^NH2^	D: 10–20	27.7 ± 0.5
CuO^COOH^	D: 10–20	−7.3 ± 0.5
CuO^PEG^	D: 10–20	−16.8 ± 0.4
TiO_2_s^core^	D: <4 *	21.5 ± 0.5
TiO_2_s^NH2^	D: <4 *	14.2 ± 0.9
TiO_2_s^COOH^	D: <4 *	21.2 ± 1.3
TiO_2_s^PEG^	D: <4 *	41.1 ± 1.5
TiO_2_r^core^	L: 56.5 ± 25.5/D: 4–15	16 ± 1
TiO_2_r^NH2^	L: 94.8 ± 43.5/D: 4–15	25 ± 3
TiO_2_r^COOH^	L: 77.7 ± 46.6/D: 4–15	−23 ± 1
TiO_2_r^PEG^	L: 69.9 ± 26.7/D: 4–15	26 ± 2
Ag^NR3+^	D: 2–8 and 50–110	44.6 ± 1.6
Ag^COOH^	D: 5–15	−32.5 ± 1.1
Ag^PEG^	D: 2–5,7–15, and 200–100	−10.7 ± 0.4
Au5^NR3+^	D: 1–8	49.0 ± 0.8
Au5^COOH^	D: 1–4	−28.8 ± 0.4
Au5^PEG^	D: 2–6	−16.5 ± 3
Au20^NR3+^	D: 9–20	42.8 ± 10
Au20^COOH^	D: 10–20 and 32–54	−26 ± 3
Au20^PEG^	D: 10–18	−37 ± 3
ND^NH2^	D: <4 *	12 ± 1
ND^COOH^	D: <4 *	−18 ± 1
ND^PEG^	D: <4 *	−5 ± 1

^#^Note, the zeta potential measurement has a size detection limit of 3.8 nm and the values for NDs and spherical TiO_2_ are at the limits of the instrument with respect to primary particle size. The zeta potential values are shown for completeness. Also, to facilitate measurements, zeta potentials were measured in 10 mm NaCl for MWCNT, NDs, and TiO_2_ rods. TiO_2_ spheres were measured at pH 3–4, and CuO at pH 5, in ultrapure water respectively to avoid precipitation at the point of zero charge.

**Figure 1:**
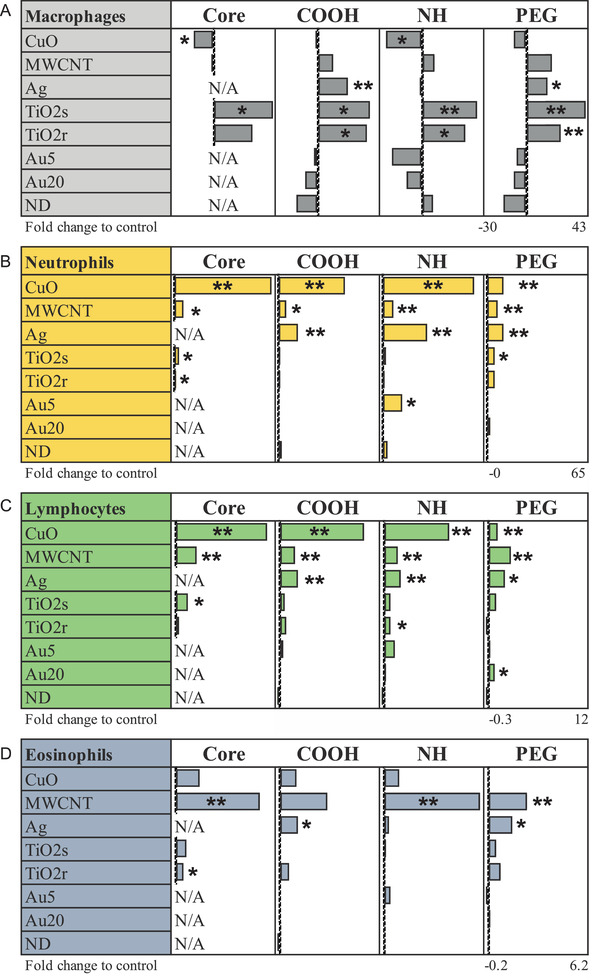
Oropharyngeal aspiration of different ENM induce changes in cell composition of bronchoalveolar lavage (BAL) fluid in mice on day 5. Especially, TiO_2_‐ENM upregulates the number of monocytes, whereas MWCNT induces enhancement of eosinophils, and CuOs neutrophils and lymphocytes in BAL fluid. *P ≤ 0.05, **P ≤ 0.01, ***P ≤ 0.001 (Two‐tailed, nonparametric Mann−Whitney U). All cell counts are counted from MGG‐stained, cytospinned slides and each group is compared to the appropriate vehicle‐treated control group. BAL cell counts are presented as normalized counts against the corresponding control cell counts. Sample size *n* = 5–8 mice per treatment group.

To identify the type of airway inflammation after exposures to 28 ENM, inflammatory cells, namely macrophages, neutrophils, lymphocytes, and eosinophils, were identified and quantified from the bronchoalveolar lavage (BAL) fluid. Significant particle‐specific changes in immune cell infiltration were observed for all four cell types (**Figure** **1**). A substantial increase in macrophage counts was seen after exposures to both spherical (TiO_2_s) and rod‐shaped Titanium dioxide (TiO_2_r) with all different functionalizations (Figure 1A). Although this suggests only a minor effect of chemistry, macrophage influx with TiO_2_r is slightly milder than with TiO_2_s, proposing that the TiO_2_ particle size and shape might have an additional effect on innate immune responses. COOH‐ and PEG‐functionalized Ag nanoparticles also triggered a significant increase in the number of macrophages in BAL fluid, while both unmodified and NH‐functionalized CuO nanoparticles significantly reduced the number of macrophages when compared to untreated control samples.

A few neutrophils and lymphocytes were found in healthy lungs, but an increase in neutrophil infiltration was related to inflammation and to acute/chronic pulmonary diseases.^[^ ^16^, ^17^ ^]^ Neutrophil infiltration and its resulting by‐products such as peroxidases and proteinases can severely damage the lung tissue. Particularly CuO, and to a lesser extent also Ag and MWCNT, were found to induce strong neutrophil and lymphocyte recruitment in the BAL (Figure 1B,C). Out of the other particles, TiO_2_s and TiO_2_r, especially their core forms, and NH‐functionalized Au5 induced slight but significant increase in neutrophil numbers when compared to controls. The toxicity of CuO and Ag could arise from the release of toxic ions from the materials and/or oxidative stress through production of ROS.^[^ ^18^, ^19^, ^20^, ^21^ ^]^ However, free ion toxicity in the BAL seems unlikely as the maximum dissolution rates of the CuO material are <0.4% (3.67, 2.00, 1.81 and 3.71 µg min^–1^ from 800 µg of metal for CuO^core^, CuO^NH2^, CUO^COOH,^ and CuO^PEG^ respectively, see Vassallo et al. 2018. Nonetheless, irritation of sensory nerves during Cu exposure is known to stimulate both adrenergic pathways and cortisol secretion, leading to a wider stress response and inflammation.^[^ ^22^ ^]^ In the case of the Ag materials, metal dissolution was below detection limit (data not shown), and regardless, any dissolved Ag released would rapidly form insoluble chloride complexes in physiological saline.^[^ ^23^ ^]^ Of course, it is theoretically possible for a vehicle effect where the intact particles release a small amount of free metal ion at the cell surface of the neutrophils and lymphocytes. In case of overall neutrophil counts from BAL, the core and NH‐ functionalizations caused the strongest neutrophil influx (Figure 1B). In reverse, PEGylated CuO and Ag, exhibiting milder, although still significant, increase in neutrophil and lymphocyte counts. We have previously reported comparable outcomes in mouse model of allergic airway inflammation with diminished response to CuO^PEG^.^[^ ^7^ ^]^ Almost identical to the neutrophilic response, lymphocyte infiltration to lungs was activated by CuO, MWCNT, and Ag (Figure 1C). Eosinophil infiltration instead, was extensively increased by MWCNT, especially by its core and aminated forms while other materials, including Ag and CuO, demonstrated only slight increase in eosinophil numbers (Figure 1D). Among others, we have previously reported eosinophilic inflammation after exposure to rigid and long MWCNT.^[^ ^11^, ^12^ ^]^ Nonetheless, the eosinophilic Th2‐type response seems to be material specific, since not all MWCNT were activating similar responses.^[^ ^24^ ^]^ The fiber paradigm further suggests that the effect is related to particles high aspect ratio and is dependent on several factors such as diameter, rigidity, and length.^[^ ^25^ ^]^ These, in turn, lead to frustrated phagocytosis, disturbed clearance (biopersistency), and damage of the lung tissue.^[^ ^11^, ^12^ ^]^

Histological assessment revealed the most pronounced inflammation in CuO‐exposed group, consisting of macrophages associated with some degree of neutrophils. No major effect was observed with CuO^NH2^, while carboxylation showed diminished reaction when compared to the CuO^CORE^. CuO^CORE^ was also revealing nuclear dust (karyorrhexis), which is likely related to leukocyte disruption (Figure S2A, Supporting Information).^[^ ^7^, ^26^ ^]^ As observed also in earlier studies, PEGylation was able to diminish the inflammatory response to CuO (Figure S2B, Supporting Information).^[^ ^7^ ^]^ MWCNT displayed a typical reaction involving CNT aggregates, perivascular and peribronchial lymphocytic infiltrates and some eosinophil infiltrates (Figure S2C,D, Supporting Information).^[^ ^11^ ^]^ Interestingly, periodic acid‐Schiff (PAS) positivity was almost completely absent in the MWCNT^CORE^‐exposed lungs (Figure S2E, Supporting Information), while seemingly more pronounced in the MWCNT^NH2^ (Figure S2F, Supporting Information). Carboxylation instead, decreased the reaction of MWCNT, as also seen from non‐significant eosinophil counts in BAL (Figure 1). Mild, or undetectable histological responses were observed in TiO_2_r and TiO_2_s. The rod shaped TiO_2_ particles were detectable in lung macrophages, but interestingly, PEGylation seemed to decrease the material detectability. Rod‐shaped TiO_2_ has been previously reported to cause pulmonary alveolar proteinosis,^[^ ^27^ ^]^ however, proteinosis was not observed from the TiO_2_r exposed lung tissue in the current study.

In summary, we could identify material‐specific cell recruitment to the BAL fluid in mouse lungs. Spherical and rod‐shaped TiO_2_ activate macrophage BAL influx regardless of their functionalization. CuO is a strong inducer of prominent neutrophilic and lymphocyte influxes. Eosinophilic inflammation dominates MWCNT‐induced response. Ag induces infiltration of multiple immune cell types, including neutrophils, lymphocytes, and eosinophils, although histological changes were not detected. Nanodiamond, although visible in the lung tissue, and Au particles, do not exert significant activation or suppression of any of the cell types studied nor changes in the lung tissue. In terms of the surface types, PEGylation dampens inflammatory cell influx when compared to COOH and NH chemistries. However, BAL counts suggest that the underlying strong toxicity of the core chemistry cannot be concealed through protective functionalization.

### 2.2 ENM Cause Distinct Transcriptional Changes in the Airways

Gene expression is fundamental for maintaining the homeostasis of the essential molecular machinery by dynamically adapting to environmental stimuli. Hence, characterizing the molecular signatures related to ENM exposure is necessary to understand the underlying biological mechanisms of adaptation, as well as the unique or shared mechanisms of toxicity.

**Figure 2:**
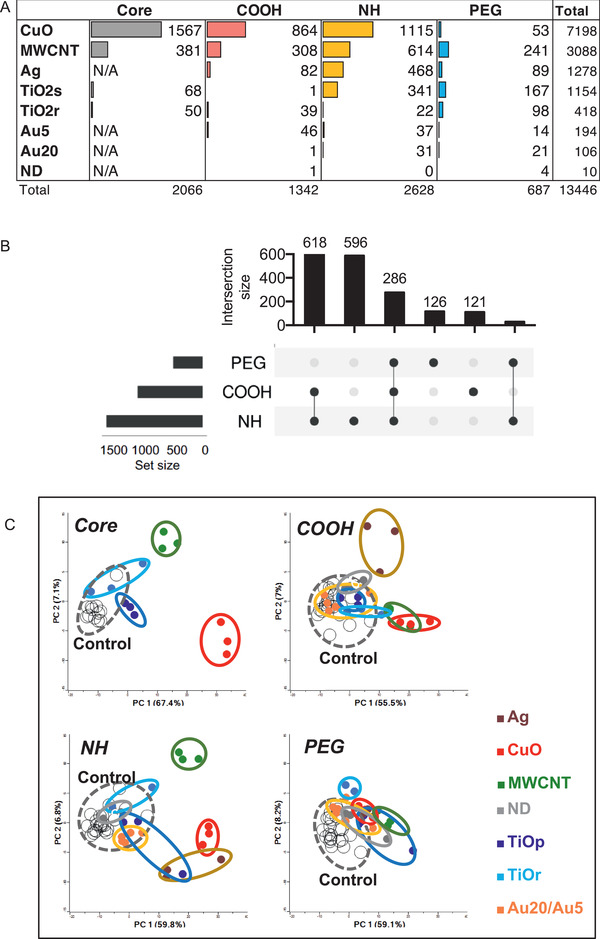
Different ENM cause varying numbers of differentially expressed genes (DEG) in mouse lungs, and changes the distribution of exposed samples in PCA. A) The number of DEG based on different ENM chemistries is the largest for the core and aminated (NH) ENM. B) Among the functionalizations, NH‐materials have the greatest number of DEG. Carboxylated (COOH) and the pegylated (PEG) ENM have the lowest number of DEG. The number of DEG based on ENM functionalization is shown in the Upset plot. C) The effects of different functionalizations are also demonstrated in PCAs. Genes were considered significantly differentially expressed with a fold change >|1.5| and Benjamini & Hochberg adjusted *p* < 0.05.

**Figure 3:**
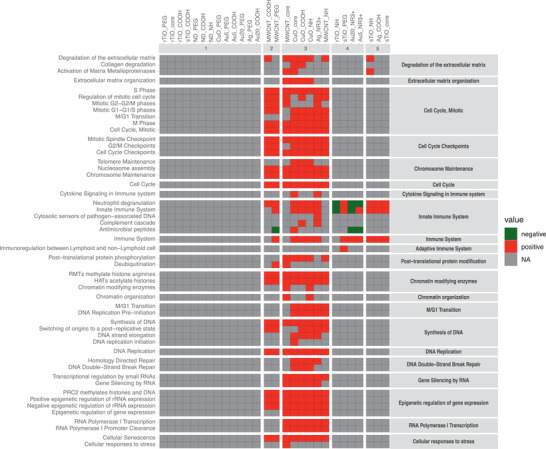
REACTOME pathway analysis shows the greatest pathway enrichment after exposure to CuO and MWCNT. Minimum of three genes per pathway were clustered by similarity (jaccard, complete linkage) and by materials at the second REACTOME level, *p* < 0.05.

In order to identify the most biologically relevant transcriptomic changes, we compared the gene expression profiles from exposed mice lung and corresponding controls and implemented a cut‐off of 1.5‐fold change at a maximum FDR of 5%, for a gene to be considered as significantly differentially expressed (DEG). In line with the BAL cell counts, gene expression profiling revealed that CuO (3599 DEG) and MWCNT (1544 DEG) triggered the most drastic changes in the transcriptome amongst all the studied 28 ENM (**Figure** **2**A and Figure S1A, Supporting Information). Next, intermediate changes in gene expression levels were observed with Ag (639 DEG), TiO_2_s (577 DEG), and TiO_2_r (209 DEG), while only modest changes were observed for Au5 (97 DEG), Au20 (53 DEG), and ND (5 DEG) particles (**Figure** **3**A). It is also evident that PEGylation caused drastically less gene expression changes compared to other functionalizations.

The core particles induced the highest number of DEG when compared to their derivatives (Figure S3B, Supporting Information). Of the functionalizations, NH‐group induced the most changes in DEG number, followed by carboxylation and then PEGylation (Figure 2B). When all the 28 particles were compared, NH‐functionalizations induced changes in a set of unique DEG (596) and additionally shared the largest number of DEG with COOH groups (618) (Figure 2B and Figure S3A, Supporting Information). PEGylated materials shared only 286 DEG with NH and COOH suggesting its lesser reactivity in in vivo settings. We next studied the similarity of the DEG between distinct ENM. Principal component analysis (PCA) (Figure 2C) showed especially NH functionalized CuO, MWCNT, and Ag clustering separately from the controls. However, much less difference was seen between controls and PEG functionalized materials (Figure 2C).

Based on the DEG counts and the PCA similarities (Figure 2 and Figure S4, Supporting Information), functionalization weakens the particle reactiveness compared to core material. Furthermore, amination causes only a slight attenuation of gene expression, and in some cases, it even enhances the particle reactivity, especially after exposure to MWCNT and Ag. This suggests that amination has a significant effect on how the particles are sensed in the airways. In reverse, carboxylation and especially PEGylation reduced the particle reactivity as identified by the number of DEG. The most dramatic difference between functionalizations is seen with CuO, where 1567 DEG of CuO^core^ was diminished to only 53 DEG in CuO^PEG^. This observation is in line with the results obtained on BAL fluid cell counts and neutrophil infiltration, as well as in the previous study by Ilves et al.^[^ ^7^ ^]^ On the other hand, when the core chemistry is non‐toxic or inert, the functionalization alone is not able to induce drastic changes, as observed with Au and nanodiamond particles and their functionalized derivatives. Indeed, nanodiamonds are intensely studied and utilized in biomedical applications due to their chemical inertness and minimal toxicity.^[^ ^26^, ^27^ ^]^

### 2.3 ENM Exposure Potency Reflects Inflammatory Cell Infiltration

BAL is a widely used diagnostic method to detect and evaluate lung related diseases.^[^ ^28^, ^29^ ^]^ Transcriptional profiling is also utilized in biomarker discovery for risk assessment of chemicals and nanomaterials.^[^ ^30^, ^31^ ^]^ As observed from the BAL cell counts and the number of DEG, the cellular and molecular responses seem to have similar trend: the more BAL cells, the more expression changes (DEG count). Thus, we further tested the associations between BAL cell counts, the number of DEG, and nanomaterial zeta potential by statistical analyses (**Table** **2**). A modest correlation (r: 0.56) between the total number of BAL cells and the total number of DEG was observed, but a very strong correlation (r: 0.91 and r: 0.91) was evident between the total number of DEG and the number of neutrophils and lymphocytes, respectively. Correlation between BAL cell number and number of DEG suggests that the more cells recruited, the more signaling cascades are activated, supporting a prominent role of the inflammatory response due to EMN exposure. The zeta potential of ENM did not significantly correlate with BAL cells or DEG counts.

**Table 2:** Cell numbers correlate along DEG. Pearsson correlation analysis shows that the number of neutrophils and lymphocytes is highly correlating with the number of up‐ and down‐regulated genes

	Z.potential	DEG_up	DEG_down	DEG_total	BALcells_total	MQ	Neutrophils	Eosinophils	Lymphocytes
Z.potential	1.00	−0.18	−0.25	−0.22	0.06	0.15	−0.06	−0.24	0.10
DEG_up	−0.18	1.00	0.93	0.98	0.74	−0.14	0.94	0.10	0.36
DEG_down	−0.25	0.93	1.00	0.98	0.66	−0.09	0.85	−0.10	0.08
DEG_total	−0.22	0.98	0.98	1.00	0.72	−0.12	0.91	0.00	0.23
all_cells_total	0.06	0.74	0.66	0.72	1.00	0.47	0.71	0.06	0.40
MQ	0.15	−0.14	−0.09	−0.12	0.47	1.00	−0.27	0.01	−0.26
Neutrophils	−0.06	0.94	0.85	0.91	0.71	−0.27	1.00	−0.03	0.55
Eosinophils	−0.24	0.10	−0.10	0.00	0.06	0.01	−0.03	1.00	0.25
Lymphocytes	0.10	0.36	0.08	0.23	0.40	−0.26	0.55	0.25	1.00

In order to uncover differences or similarities in the perturbed biological functions associated with ENM exposures to mice lungs, pathway enrichment analyses were performed on identified DEG (Figure 3 and Figure S5, Supporting Information). The most pronounced outcome at the pathway‐level was appreciated after CuO and MWCNT exposures (Figure 3). Reactome pathways such as signal transduction, cell cycle, DNA replication, and gene expression (transcription) were found in common between all these materials (Figure S5, Supporting Information). Negative activation of biological oxidation‐pathway was unique to CuO^CORE^ and CuO^NH2^. On the other hand, CuO^PEG^ clustered into a separate group together with the materials displaying no significant pathway activation (Figure 3), again, highlighting the diminishing effect of PEGylation. DNA repair was found enriched after Ag^NR3^, all CuO^CORE^, CuO^COOH,^ and CuO^NH2^ exposures (Figure S5, Supporting Information), suggesting that this set of ENM are able to damage the genetic material either by direct contact with internalized particles (released ions) or due secondary effects like metabolic by‐products. In addition, particle access to the nucleus and secondary genotoxic effects due to inflammation may play a role.^[^ ^8^ ^]^ Functionalized MWCNT were clustering together with Ag^NR3^ showing activation of many common pathways with CuO‐cluster, but instead, were not inducing DNA repair‐ or ECM‐related pathways as most of the CuO particles (Figure 3). Ag ions are known to be toxic to microorganisms and thus, are utilized in biomedical applications such as plasters and bandages. In line with this, we reported recently that Ag nanoparticles reduced the secretion of pro‐inflammatory cytokines in response to LPS, likely as a result of the release of Ag ions leading to an interference with TLR signaling.^[^ ^32^ ^]^ Based on the DEG counts in Ag‐exposed lungs, Ag^NR3^ was inducing the strongest changes in gene expression with 377 upregulated and 91 downregulated genes. COOH and NH_3_ functionalized Ag both induce neutrophil degranulation and immune system pathways (Figure 3 and Figure S3, Supporting Information), whereas Ag^PEG^ was clustering together with other non‐responsive materials, and showed no enriched pathway activation. Among Au nanoparticles, only NH_3_ functionalization seemed to exert enrichment of pathways through negative activation of neutrophil degranulation and antimicrobial peptides (Figure 3).

Overall, the pathway activation and similarity‐based clustering reflect the severity of the inflammation observed also by the inflammatory BAL cell counts. The results suggest that based on the activated Reactome pathways, ENM can be roughly separated at least into three groups: the non‐reactive, reactive, and hazardous materials. The non‐reactive group included materials with COOH‐ and PEG ‐functionalizations, as well as TiO_2_r^CORE^, but no NH_3_ functionalization.

### 2.4 Biological Responses to ENM Exposure is Associated with Particle Shape

**Figure 4:**
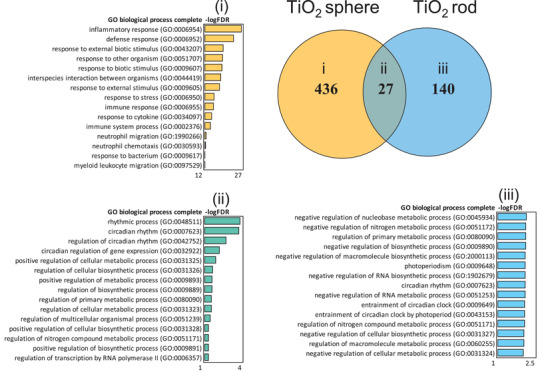
TiO_2_ spheres induce more DEG in mouse lung tissues than TiO_2_ rods when all the DEG from different functionalizations is combined. Only 27 common genes overlap between the two tested TiO_2_ ENM in the Venn diagram. TiO_2_ spheres activate inflammation‐related GO biological processes. TiO_2_ rods instead, activate metabolic and biosynthetic processes.

The DEG from the two differently shaped titanium nanoparticles, spherical (TiO_2_s) and rod (TiO_2_r), were compared to recognize the shape‐related differences in responses. Although significant macrophage influx in lungs was comparable with both TiO_2_ materials and the underlying chemistry (TiO) was the same, the total number of DEG between spherical and rod was distinctive (436 and 140, respectively), with only 27 overlapping genes (**Figure** **4**). These differences in DEG counts suggest that the airway response to TiO_2_ materials is related to the particle shape and size. At the pathway level, spherical TiO_2_ particles enriched several immune‐related GO biological processes, such as inflammatory and defense responses, response to cytokine and neutrophil or leukocyte migration. Rod‐shaped TiO_2_, instead, triggered much milder responses related mostly to negative regulation of RNA and metabolic processes. The shared 27 genes were enriched especially circadian rhythm related pathways (Figure 4). The primary size of the spherical TiO_2_ particles is <4 nm, whereas the diameter of rods varies between 4 and 15 nm, and length from 31 to 138 nm (Table 1). This leads to a notion, that smaller and larger particles are sensed and processed in distinct manner in the airways. In addition, the smaller particles might more easily interact with other cell types such as airway epithelial cells, and due to the extremely small size, also reach distinct areas inside the cell. There is a postulation of ≈100 nm cut‐off in distinct cellular uptake mechanisms, suggesting that with the larger rods, phagocytosis or macropinocytosis is required instead of the endocytic pathways observed with smaller (<100 nm) particles.^[^ ^33^ ^]^

The transcriptional patterns of the two different sized Au nanoparticles were also compared to understand the differences in size‐related biological responses (Figure S6, Supporting Information). Generally, all Au particles in this study were causing relatively few transcriptional changes. We were not able to identify statistically significant pathways enriched by GO Biological Processes and therefore studied their Molecular Functions by Ingenuity Pathway Analysis (IPA). As with the TiO_2_ particles, also the smaller Au particles (5 nm) appeared to trigger more noticeable defense responses than the 20 nm Au particles, elevating pathways such as cellular function and maintenance, cell morphology, and cell cycle. The larger, 20 nm particles stimulated mainly genes related to cell morphology and cell death and survival (Figure S6, Supporting Information). It should be noted, however, that the expression changes were very modest, suggesting no severe airway inflammation and consequent toxicity of either Au particles, as also supported by the minor changes in BAL cell influx.

### 2.5 Pathway Enrichment Reveals Common and Functionalization‐Specific Responses

**Figure 5:**
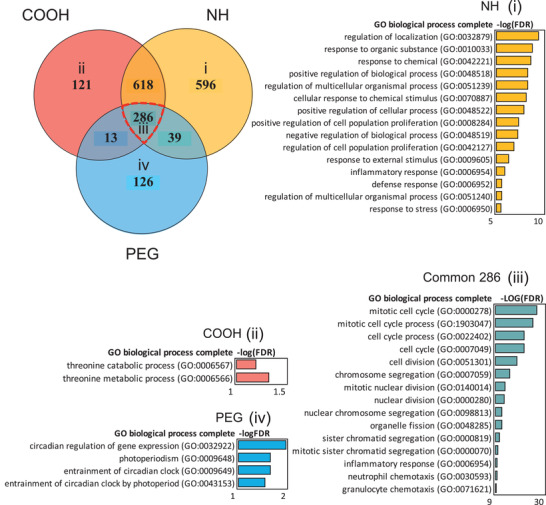
Aminated, carboxylated, and pegylated ENM show different biological functions in VENN diagram. NH functionalization shows clear enrichment of chemical stimulus related biological processes in Panther, whereas only minor enrichment is seen in COOH‐specific, or PEG‐specific DEG. The strongest enrichment of cell cycle related processes can be seen in the common 286 DEG. The FDR‐corrected Fisher's Exact *p*‐values are shown as bars corresponding to the color of functionalization.

To recognize the effect of the functionalizations, all the DEG from each surface chemistry were arranged into three groups: “NH” “COOH,” and “PEG.” This resulted in 121 specific DEG in COOH group, 126 PEG‐specific DEG, and 596 NH‐specific DEG and shared 286 DEG (**Figure** **5**). Based on the consequent Gene Ontology enrichment of the specific groups, functionalizations were stimulating distinctive biological processes despite the underlying particle chemistries.

Based on the BAL counts, amination was associated with enhanced neutrophil recruitment as well as to strongly increased number of DEG. Those genes are enriching the regulation of localization, response to organic substance, and response to chemicals in GO biological processes (Figure 5). The latter two belong to the sub‐category of cellular response to IL‐18, a proinflammatory cytokine enhancing the inflammation in tissue. When the upstream cytokine regulators of these genes were studied by IPA, the proinflammatory TNF, IL‐1*α*/*β*,IFN‐*γ* and IL‐6 could be identified (Figure S7, Supporting Information). Other cytokines such as IFN‐*γ* is specific for the differentiation of Th1‐cells, and IL‐4 is specific for differentiation of Th2 cells. Therefore, it can be hypothesized, that aminated nanoparticles are capable of inducing both innate and adaptive immunity pathways. Genes activated by COOH enrich threonine catabolic and threonine metabolic processes, which play a potential role in ATP production and pH regulation (Figure 5). PEGylation is exerting circadian regulation of gene expression and photoperiodism that are related to the time‐associated changes of gene expression in organisms (Figure 5).

The different functionalizations share 286 (15.9%) common DEG playing a role in cell division with very significant FDR values (up to 8.82E‐28) (Figure 5). The identified pathways include mitotic cell cycle, mitotic cell cycle process, and cell division. It could be speculated, that nanomaterial exposure causes cell death in the lungs, leading to enhanced cellular replacement, observed as enhanced cell division. The involved DEG were regulated upstream by colony stimulating factor,CSF2, which plays a very important role in the macrophage differentiation and function, and several proinflammatory cytokines including IL‐6, TNF, IL‐17A, and IL‐1*β*, which are known to orchestrate acute immune responses (Figure S7, Supporting Information).

Altogether, the results strongly imply that in terms of functionalization, amination activates the strongest inflammatory response among the three distinct functionalizations. Nonetheless, all the functionalizations are able to induce immune system activation as noted from the Gene Ontology terms enriched by the common DEG, but do not necessarily imply strong, long‐lasting immunotoxic consequences.

### 2.6 PEGylation Modulates Particle Toxicity by Suppressing ENM‐Induced Inflammation and DNA Damage

**Figure 6:**
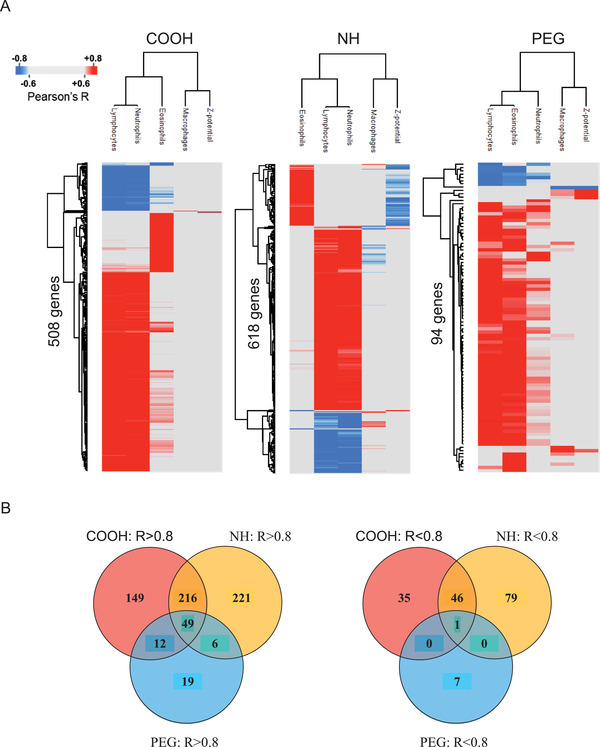
DEG from functionalized ENM correlate with the BAL cell counts and corresponding zeta potential of particles. A) The strongest correlations (Pearson r ≥ |0.8|) are identified for lymphocytes and neutrophils, with both COOH (508 DEG) and NH (618 DEG) ENM, and in lesser amount with PEG (94 DEG), which also showed association to eosinophils. B) Most of these DEG were positively correlated (r ≥ 0.8) and 60% (510 genes) of all immune cell count‐associated genes (r ≥ |0.8|) are unique to a specific functionalization.

In this study, a strong association (r: >0.90) between immune cell infiltration and the number of DEG was observed (Table 2). Particle surface chemistry (COOH, NH, or PEG) have also an effect on the magnitude of the nanomaterial‐induced transcriptomic response (Figure 2B and Figure 5). Thus, we propose, that by investigating the correlation between ENM‐induced immune cell infiltration and associated DEG, the primary genes that underpin the effect of functionalization on toxicity can be identified. For this, the DEG were split into three groups comprising particles with either NH‐, COOH‐ or PEG‐based surface functional groups. Exposure‐specific BAL cell counts and zeta potentials of the corresponding particles were correlated to the exposure‐specific DEG (**Figure** **6**).

The strongest correlation (r: ≥ |0.8|) to functionalization‐induced changes in gene expression were identified for lymphocytes and neutrophils, which were jointly associated to the expression of 508 DEG in mice exposed to COOH‐functionalized particles, as well as to 618 DEG in exposures to particles with NH‐functionalized surfaces. In mice exposed to PEGylated particles, similarly strong correlations were identified between 94 genes and the number of infiltrated eosinophils and lymphocytes. Whilst the number of infiltrated eosinophils, lymphocytes, and neutrophils were strongly associated with particle surface chemistry, only a handful of genes showed notable association (r: >|0.6|) to either macrophage infiltration or particle zeta potential (data not shown). Heatmaps based on the correlation score between the expression of functionalization‐associated genes and immune cell counts/particle zeta potential are shown in Figure 6A. Most (80%) of these genes were positively correlated and 60% (510 genes) of all immune cell count associated genes were unique to a specific functionalization (Figure 6B). 300, 184 and 26 immune cell associated genes were unique to either the NH, COOH, or PEG functionalization, respectively. Overlapping genes, mostly amongst the positively correlated, were also identified (Figure 6B).

In terms of the underlying mechanisms, the most significantly enriched biological processes were identified from the 50 shared DEG that were strongly associated to cell levels of neutrophils, lymphocytes, and eosinophils, exposed to particles with either NH, COOH, or PEG functionalization (Figure S8, Supporting Information). The top enriched biological processes were related to mitotic cell cycle and chromosome organization. Amongst the genes that were associated to immune cell infiltration levels in response to one specific surface chemistry, COOH‐functionalized nanomaterials triggered the most significant pathways, with lymphocyte and neutrophil migration identified as the most enriched biological processes. NH‐specific BAL cell count‐associated genes were predominantly involved in regulation of DNA replication. PEG‐specific immune cell‐associated genes were involved in even fewer biological processes. The top enriched pathways were regulation of protein kinase activity with relevance to binding of DNA by transcription factors. Interestingly, pathways suggestive of DNA damage response were identified only in the top enriched pathways from COOH‐ and NH‐responsive genes. In general, significantly smaller pathway fold‐enrichment scores were identified for functionalization‐specific DEG with strong association to immune cell counts, compared to the DEG which were shared between all three functionalizations.

The identified changes in genes related to cell division, chromosomal organization, and DNA replication (probably a direct outcome of the cellular cytoxic effects of these nanomaterials), occur irrespective of the ENM surface chemistry. Nonetheless, PEGylation appears to suppress nanomaterial toxicity by decreasing the magnitude of particle‐induced changes in cell division, chromosomal organization, and DNA conformation changes (Figure S8, Supporting Information). Besides direct toxicity, functionalization can be expected to affect cellular uptake of the particles, as observed in in vitro studies for Au NPs.^[^ ^34^ ^]^ Taken together, surface modification has varying effects on the airways but the toxicological responses also depend on the underlying core chemistry.

### 2.7 The Expression Signature Derived from a Subset of 50 DEG Associated with Cellular Infiltration Generates Three Clusters of ENM exposures

**Figure 7:**
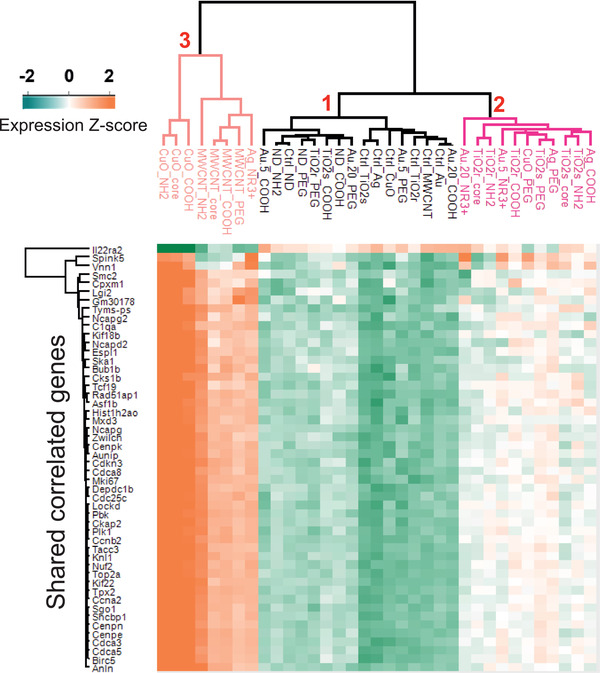
Heatmap and hierarchical clustering of highly positively and negatively correlating common 50 DEG with BAL fluid cells are dominating in the inflammatory cell responses while the functionalizations have only minor effects. Based on the Pearson correlation, three main clusters are emerging (1–3). The cluster 1 contains the mildly toxic ENM (TiO_2_r^PEG^, TiO_2_s^COOH^, Au5^PEG^, Au5^COOH^, Au20^PEG^, Au20^COOH^, ND^COOH^, ND^NH2^, and ND^PEG^) and the negative control samples. The cluster 2 involves the moderately toxic nanomaterials, consisting of Au5^NR3+^, Au20^NR3+^, TiO_2_r^Core^, TiO_2_r^COOH^, TiO_2_r^NH2^, TiO_2_s^Core^, TiO_2_s^NH2^, TiO_2_s^PEG^, Ag^COOH^, Ag^PEG^, and CuO^PEG^. And last, the third cluster contains the most toxic ENM including Ag^NR3+^, CuO^Core^, CuO^COOH^, CuO^NH2^, MWCNT^Core^, MWCNT^PEG^, MWCNT^COOH^, and MWCNT^NH2^.

Based on functionalization‐specific DEG categorization, as well as correlation to level of immune cell infiltration, we identified 50 primary response genes whose expression was modified by all materials irrespective of the surface chemistry. To understand whether the expression of these genes alone could rank the tested particles according to their health hazard potential, we plotted a heatmap of the average relative expression of these genes across all exposed and unexposed sample groups (**Figure** **7**). Three main clusters emerged:

**CLUSTER 1**–comprising TiO_2_r^PEG^, TiO_2_s^COOH^, Au5^PEG^, Au5^COOH^, Au20^PEG^, Au20^COOH^, ND^COOH^, ND^NH2^, and ND^PEG^, all clustered together with the control samples.

**CLUSTER 2**–consisted of Au5^NR3+^, Au20^NR3+^, TiO_2_r^Core^, TiO_2_r^COOH^, TiO_2_r^NH2^, TiO_2_s^Core^, TiO_2_s^NH2^, TiO_2_s^PEG^, Ag^COOH^, Ag^PEG^, and CuO^PEG^.

**CLUSTER 3**–consisted of the most toxic nanomaterials amongst all 28 ENM—Ag^NR3+^, CuO^Core^, CuO^COOH^, CuO^NH2^, MWCNT^Core^, MWCNT^PEG^, MWCNT^COOH^, and MWCNT^NH2^.

Interestingly, the clusters generated by this core set of 50 genes, reflect the toxicity of each particle as observed from the extent of cellular infiltration (Figure 1) and the number of DEG (Figure 2A). PEG‐functionalized ENM, generally less toxic than either the COOH‐ or NH‐functionalizations, were predominantly (with the exception of TiO_2_s^PEG^) represented in Cluster 1. Cluster 3 was highly enriched for the most toxic nanomaterials amongst all 28 ENM. Incidentally, in addition to cellular infiltration and number of DEG, Cluster 3 ENM were also the most cytotoxic ENM observed in in vitro exposures of human primary macrophages and undifferentiated human THP‐1 cell lines.^[^ ^35^ ^]^ MWCNT were also exceptional, as all of its four surface chemistry types (Core, PEG, COOH, and NH) clustered in the “most toxic” Cluster 3 ENM group. It seems that the greater the toxicity of the core material, the ability of a functional group modification to affect the materials’ toxicity decreases.

## 3 Conclusion

Eight different ENM functionalized with three different surface modifications, totaling up to 28 distinct ENM were examined by repeated, 4‐day oropharyngeal aspiration exposures using mouse as a model organism. This extensive data set comprises cytological, histological, and transcriptional analyses and thus, provides the largest available in vivo data set to date. Our results imply that surface modification has varying effects on the airways but those toxicological responses also depend on the underlying core chemistry.

While BAL cell quantification and histological evaluation are a reasonable and consistent initial step to evaluate the severity of the ENM‐induced immune responses on the airways, assessing the global transcriptome provides more detailed knowledge about the specific responses and distinct biological processes. This further enables a detailed mapping of the molecular events consequently leading to the toxic, and possible long‐term outcomes. The performed histological and cytological evaluation revealed CuO, Ag, and MWCNT as the most toxic ENM among the tested panel in terms of strong neutrophilic and eosinophilic cell influx, nuclear dust formation, and mucus hypersecretion. Moreover, comparable, increased macrophage influx due to TiO_2_ nanomaterials with distinct shapes was observed.

Transcriptional analyses further completed the observed outcomes in terms of gene activation (number of DEG) but only more detailed transcriptional analyses allowed to dissect specific, functionalization‐, and size/shape related responses. Transcriptomics experiments can be used to underpin molecular level changes, and thus, to scrutinize more specific responses associated to distinct ENM properties. This instead, provides great advancements to toxicological evaluation in terms of read‐across and adverse outcome pathways.^[^ ^1^ ^]^ As transcriptional level responses are depicting also the subtle changes not visible in cytology or histology, ethically more reasonable exposure strategies such as lower exposure doses will become achievable.

We strongly advocate to complement classical toxicity studies based on the observation of apical endpoints with more thorough examination of cellular and molecular events namely mode/mechanisms of action leading to possible toxic outcomes. For this, transcriptomics and toxicogenomics provide great opportunities for future toxicity evaluation and assessment, helping to create deeper understanding and predictive models eventually speeding up the toxicity assessment of ENM.

## Experimental Section

##### Panel of Nanomaterials

Nanomaterials were provided by the FP7‐NANOSOLUTIONS consortium. ENM synthesis, functionalization, and characterization are described in detail in the previous publications.^[^ ^35^, ^36^ ^]^ In short, CuO particles were synthesized by the precipitate decomposition method. MWCNT^CORE^, MWCNT^NH2^, and MWCNT^COOH^ were produced via catalytic carbon vapor deposition. For the MWCNT^PEG^, a noncovalent functionalization process was used. Au5 and Au20 particle synthesis was performed by growing the particles with self‐assembled thiol monolayers on the growing nuclei. Ag particles were prepared by reduction of Ag^+^ to Ag^0^ with excess NaBH_4_ solution. TiO_2_s were prepared by hydrolysis of titanium tetrachloride solution whereas TiO_2_r core was obtained by forced hydrolysis in acidic conditions. Finally, nanodiamonds were prepared by detonation. ENM characterization was described earlier by Gallud et al.^[^ ^35^ ^]^ Briefly, ENM were characterized by DLS and zeta potential measurements using a Malvern Zetasizer Nano ZS (model ZEN3600) with a 632.8 nm laser wavelength. The Zetasizer v.6.32 software was used for data processing. UV–vis measures were accomplished on a UV–vis spectrometer Lambda 750 (Perkin Elmer). The UVWinLab software was utilized for data processing. Raman spectroscopy measurements were performed using a WITec alpha 300 RAS device, and a laser operating with the WITec project FOUR software. Impurities were investigated by inductively coupled plasma‐mass spectrometry. The presence of functional groups was confirmed with XPS (Table S1 and Figures S9–S15, Supporting Information). XPS experiments were performed in a SPECS Sage HR 100 spectrometer with a non‐monochromatic X ray source (Magnesium K*α* line of 1253.6 eV energy and a power applied of 250 W and calibrated using the 3d5/2 line of Ag with a full width at half maximum (FWHM) of 1.1 eV. An electron flood gun was used to compensate for charging occurring during XPS data acquisition. The selected resolution for the spectra was 30 eV of pass energy and 0.5 eV step^−1^ for the general survey spectra and 15 eV of pass energy and 0.15 eV step^−1^ for the detailed spectra of the different elements. All measurements were made in an ultra‐high vacuum (UHV) chamber at a pressure around 5•10–8 bar. ENM samples were first dispersed in water and casted on pre‐cleaned Si wafers, followed by drying in a vacuum oven. For all TiO_2_ based materials ligands have been attached through silanes while for the rest of the nanoparticles functionalization took place through thiols with the exception of nanotubes and nanodiamonds that were oxidized to display carboxylate groups and further functionalized from there. Silane modification and carboxylation result in more stable bounds than thiols which could be eventually exchanged but this is a characteristic of thiol bounds

Nanomaterial stock dispersions were prepared in glass tubes, in endotoxin free water (HyClone, HyPure Cell Culture Grade Water, Thermo Scientific, Waltham, MA USA) according to the NANOSOLUTIONS standard operating procedures provided for each material. The dilutions of the working suspension were prepared into a sterile PBS in a concentration of 200 µL mL^−1^. The material handling, weighing, and suspensions were prepared in ultra clean conditions, with sterile equipment. Control samples were prepared to pure PBS, PBS + polyvinylpyrrolidine, or PBS + albumin (0.1% BSA, Sigma) corresponding to the ENM suspension.

##### Animals

Female C57BL/6 mice (7−8 weeks old) were purchased from Scanbur A/S (Karslunde, Denmark) and quarantined for 5 days and housed in stainless steel cages with aspen chip bedding, in groups of four. The housing conditions were carefully controlled with temperature (20–21 °C) and humidity (40−45%) and 12 h dark/light cycles. Mice received food and water ad libitum.

##### Study Design and Oropharyngeal Aspiration Exposures

The study design is shown in Figure S1, Supporting Information. Mice were exposed by oropharyngeal aspiration under isoflurane anesthesia to 10 µg of ENM per day in (50 µL) of PBS for 4 consecutive days, mimicking a 1‐week exposure scenario. Control mice received a corresponding vehicle, either plain PBS or supplemented with polyvinylpyrroline (for nanodiamond particles) or albumin (for MWCNT) as described in suspension preparation section. BAL and tissue samples were collected 24 h after the final exposure.

Moderate dose of 10 µg per day was selected based on the previous studies.^[^ ^12^ ^]^ The study was approved by National Animal Experiment Board from Regional State Administrative Agency of Southern Finland (ESAVI‐3241−04.10.07−2013).

##### BAL Cell Counts and Histology

Leukocyte infiltration into mice lungs was analyzed by BAL. After sacrificing and blood collection, the trachea was instantly cannulated with a syringe and lungs were flushed with (800 µL) of PBS. BAL fluid was cytocentrifuged onto slides and May Grunwald Giemsa (MGG)‐stained BAL cells (macrophages; neutrophils, eosinophils, and lymphocytes) were counted under a light microscope (Leica DM 400B; Leica, Wetzlar, Germany) with 40× magnification. Three high‐power fields per sample were counted and the mean value was recorded. Mann–Whitney tests were used for BAL cell counts between two‐group analyses. *P‐*value <0.05 was considered as statistically significant.

For the histological evaluation, parts of the lungs were formalin‐fixed and paraffin embedded. The cut and affixed samples were stained with hematoxylin and eosin and PAS and evaluated with optical microscope.

##### RNA Extraction

After sacrificing and BAL collection, the lungs were removed, cut and transferred to RNALater stabilizing solution (Ambion, Life Technologies, CA, USA), and stored at −80 °C. Total RNA (totRNA) from the RNAlater‐stabilized lung samples was isolated and purified by phenol/chloroform isolation method according to the instructions by Bioline Reagents. In brief, tissue samples in RNALater were thawed and moved to the lysing matrix tubes with ceramic spheres (D, 1.4 mm) (MP Biomedicals, Illkirch, France), containing 1 mL of TRIsure reagent (Bioline Reagents, Ltd., London, UK). Samples were homogenized in a FastPrep FP120 homogenizer (BIO 101, Thermo savant, Waltham, MA, USA). RNA was separated with chloroform, precipitated with isopropyl alcohol, washed with 75% ethanol, re‐dissolved into DEPC‐treated water, and stored in −80 °C. Quantity and quality of the mRNA were measured with NanoDrop spectrophotometer (ND‐1000, Thermo Fisher Scientific Inc., Wilmington, NC, USA) and Bioanalyzer (Agilent Technologies, USA). Two to three samples from the same experimental group were pooled, and independent pools of samples with RIN >8.3 were used for DNA microarray analysis.

##### Microarrays

Two to three totRNA samples with good quality (RNA integrity value >7.5) were pooled as one and diluted with ultrapure, sterile water to 200 ng in 1,5 µL.

The sample was randomly labeled with Cy3 or Cy5 (Quick amp labeling kit, two‐color, Agilent). Labeled samples were unsystematically hybridized to Agilent Sure Print G3 Mouse, GE8×60K DNA microarrays according to the manufacturer's protocol (two‐color microarray‐based gene expression analysis, low input quick amp labeling, Agilent, USA). Hybridized slides were scanned with Agilent microarray scanner (DNA microarray scanner with Surescan high‐resolution technology, model G2505C, Agilent, USA), and the raw data were extracted using Agilent feature extraction software (V12.0.1.1). The data are available in Gene Expression Omnibus with the accession number GSE157266.

##### Data Analysis

###### Microarray Data Processing

Changes in gene expression were analyzed with eUTOPIA—an R‐based graphical user interface composed of standard bioinformatics packages.^[^ ^37^ ^]^ Sample pre‐processing and batch effect correction were carried out as described previously.^[^ ^7^ ^]^ Between group differential gene expression was performed by Limma model analysis, using Benjamin & Hochberg method for multivariate correction of false discovery rate. A minimum log2 difference of 0.58, and a maximum adjusted *p*‐value of 0.05 were implemented as cut‐off to consider a gene as significantly differentially expressed between exposed and unexposed mice. Genes associated with particle‐induced cellular infiltration or particle zeta were investigated via Pearson's correlation analysis. Perseus graphical user interface was used to generate clusters and heatmaps. Clustering parameters used were as follows; Distance: Euclidean, Linkage: Average and Cluster Preprocessing: K‐means.

###### Pathway Analyses

The physiological implications of the DEG identified for each exposed/unexposed contrast were predicted via biological process or disease/function enrichment analyses using either the reactome pathway database,^[^ ^38^ ^]^ or IPA (QIAGEN) pathway analysis tools, respectively. Only DEG with a fold change above 1.5 at a maximum FDR of 5%, were used for pathway enrichment analyses.

###### Statistical Analysis

Experiments were performed with *n* = 5–8 mice per treatment group. Two‐tailed, nonparametric Mann−Whitney U tests were used for BAL cell count statistics. The bar plots in Figure 1 are presented as normalized values against the control BAL counts. For microarrays, two biological samples (total RNA) were pooled as one, totaling to three replicates per treatment group. The raw intensity values from the microarray experiments were quality checked and exported to R shiny application eUTOPIA.^[^ ^37^ ^]^ Low‐quality probes were excluded using a threshold of 75% quantile of negative probes in at least 85% of the samples. Log2 transformed probe intensity values were quantile normalized. Technical variation from dye and array were removed with R‐package SVA using ComBat method.^[^ ^39^ ^]^ Probes were mapped and summarized by median values. Gene expression changes between treatment and corresponding control group were defined with R package Limma,^[^ ^40^ ^]^ using empirical Bayes pairwise comparison. Genes with a fold change >|1.5| and Benjamini & Hochberg adjusted *p* < 0.05 were considered significantly differentially expressed. Reactome pathway analysis was performed with R shiny application FunMappOne.^[^ ^41^ ^]^ Only annotated DE genes were used. A minimum of 3 genes per Pathway/Ontology was used as a threshold. Jaccard index with complete linkage was used for similarity clustering. Aggregation function “median” with correction method “fdr” were set as displaying parameters. Pearson correlation coefficient was used for the correlation analysis. GO enrichment analysis of biological processes was performed with Panther classification system with FDR corrected Fisher's exact *p*‐values.^[^ ^42^ ^]^

## Conflict of Interest

The authors declare no conflict of interest.

## Author Contributions

J.N. and M.I. contributed equally to this work. P.A.S.K. performed the ENM exposures. P.A.S.K. and M.I. sacrificed the mice, collected, and pre‐processed the samples. P.A.S.K. performed microarray analyses and statistical analyses. J.N. analyzed, investigated, and visualized the data. G.V. confirmed the dispersion protocols and performed the DLS measurements. H.N. supervised the dispersion studies. H.W. evaluated the lung histology. T.S. confirmed RNA quality by Bioanalyzer analyses. J.K. supervised the quality analyses. K.S. served as the overall coordinator of the project and provided expert advice on the toxicological studies. B.F. contributed to the analysis and interpretation of the results and participated in the manuscript writing. P.K. contributed to formal analyses, visualization, and result interpretation. D.G. conducted the statistical analyses, supervised the microarray analyses, and interpreted the results. H.A. designed the studies and supervised the experiments. P.A.S.K. drafted and P.A.S.K., J.N., P.K., and H.A. wrote the manuscript. All authors participated in manuscript evaluation and have approved the final document.

## Supporting information

Supporting Information

## Data Availability

The transcriptomics data have been deposited in the NCBI Gene Expression Omnibus (GEO) database (accession no. GSE157266). Other relevant source data are available through the data repository of FP7‐NANOSOLUTIONS (http://nanosolutions.cs.unisa.it/login.cgi).
