# Supplementary material for: Toxicogenomic Profiling of 28 Nanomaterials in Mouse Airways
Source: Adv Sci (Weinh). 2021 Mar 8;8(10):2004588. doi: 10.1002/advs.202004588 (PMC8132046; doi:10.1002/advs.202004588)
Supplement: Supplementary file 1 — Supporting Information [file ADVS-8-2004588-s001.pdf]

© 2021 Wiley-VCH GmbH

ADVANCED SCIENCE
Open Access

Supporting Information

for *Adv. Sci.*, DOI: 10.1002/advs.202004588

Toxicogenomic Profiling of 28 Nanomaterials in mouse airways

Pia A.S. Kinaret[1,2], Joseph Ndika[3#], Marit Ilves[3#], Henrik Wolff[4], Gerard Vales[4], Hannu Norppa[4], Kai Savolainen[4], Tiina Skoog[5], Juha Kere[5], Sergio Moya[6], Richard D. Handy[7], Piia Karisola[3], Bengt Fadeel[8], Dario Greco[1,2,9,10], Harri Alenius[3,8]*

Supporting information to:

Toxicogenomic Profiling of 28 Nanomaterials in mouse airways

Pia A.S. Kinaret *et al.*

Supplementary Figure Legends:

Figure S1. Schematic view of the study design indicates that female C57BL/6 mice are oropharyngeally exposed individually to 28 ENMs on four consecutive days. After 24h of the last exposure the samples are collected for cellular, histological and transcriptomic analyses.

Figure S2. Histological evaluation of A) CuO^{core}, B) CuO^{PEG}, C) $MWCNT^{CORE}$, D) $MWCNT^{PEG}$, E) $MWCNT^{CORE}$, F) $MWCNT^{NH3}$ exposures. H&E stained lung images (A-D) are shown at 400× magnification, with 50 µm scale bars. PAS-stained lung samples (E and F) are shown at 200x magnification with 100 µm scale bars. Blue arrows are depicting nuclear dust, black arrows indicate visible MWCNT, red arrows illustrate PAS positivity (mucus production) and green arrows are pointing to eosinophils.

Figure S3. Upset plots of CuO, MWCNT and TiO_2 ENM by A) particle and by B) functionalization. Only clusters with more than 30 interactions are shown.

Figure S4. PCA of all genes in ENM-exposed mice lungs shows the distribution of different exposures. The groups are indicated as an average of six biological replicates. CuO core is dominating in gene variation, and expression variation is degreased in the order of NH, COOH and PEG in the PCA.

Figure S5. Especially CuO and MWCNT show pathway enrichment at REACTOME pathway analysis at the level 1, $p < 0.05$. Moreover, enrichment was also seen in NH-derived DEG. Minimum of 3 genes per pathway are clustered by similarity (Jaccard, complete linkage) and by materials.

Figure S6. Au5 and Au20 are causing similar effects in mice and only slight enrichment of Molecular Functions is seen in pathway analyses. DEG from all functionalizations of Au5 and Au20 were combined, and unique DEG were analyzed by IPA Molecular Functions (BH corrected p-values). No significant pathway enrichments were seen for Au5 or for Au20 in Panther analyses on GO Biological Processes.

Figure S7. Different functionalizations of TiO_2s, TiO_2r, CuO and MWCNT in venn diagram and their BH corrected p-values upstream regulator analyses in IPA. Significant cytokine upstream regulators can be identified clearly only to NH-associated and shared to all DEG.

Figure S8. The number and the names of the associated GO pathways are derived from highly correlating ($r \geq |0.8|$) DEG and BAL cells (in Figure 6). Pathway enrichment is strongest in the shared 50 correlating DEG, and PEG showed the smallest enrichment of GO biological processes (Panther). The negative logarithm of corrected FDR-value is shown for each pathway per functionalization (COOH, NH, PEG).

Figure S9. XPS spectra of TiO2s^{COOH} : Ti2p (top left); O1s (top right); C1s (bottom left) and N1s (bottom right).
From Ti2p and O1s spectra, it can be seen that the ENM are mainly composed of TiO2. From C1s and O1s spectra, we can see the presence of C-H and C-C bonds, as well as C-O and C=O-OH bonds, indicating the existence of –COOH groups on the nanoparticles surface. We can also see the –N-H and C-N-H bonds in the N1s spectrum, which is also supposed to appear according to the functionalization process of the nanoparticles. ((TiO2)-CH2-CH2-CH2NHOC(O)CH2CH2COOH)

Figure S10. XPS spectra of TiO2s^{NH2} Ti2p (top left); O1s (top right); C1s (bottom left) and N1s (bottom right).
For TiO2s^{NH2} their surface chemistry is supposed to be (TiO2)-Si-CH2CH2CH2NH2 after the surface modification. As shown in the Ti2p and O1s spectra above, Ti-O bonds in Ti 2p and O1s spectra show the nanoparticles are TiO2. In C1s spectrum, the band located at 285eV can be assigned to the sp3 and sp2 C-C and C-H bonds, from the N1s spectrum, C-N bonds and NH3+ can also be found in the band ranging from 398-404eV. These results confirmed that NH3+ is present on the surface of the TiO2sNH2 nanoparticles.

Figure S11. XPS spectra of Au20COOH: C1s (top left); Au4f (top right); S2p (bottom left), O1s (bottom right).
From the Au4f spectrum, we can see that gold exists mostly in the form of Au(0) and there are possibly a small amount of Au (I) in this sample. We observe a strong peak for C=O bonds and we can also see C-O (O1s spectrum), C-C and C-H (C1s spectrum).

Figure S12. XPS spectra of Au20^{NR3}C1s (top left); Au4f (top right); S2p (middle left), O1s (middle right), Cl2p (bottom left) and N1s (bottom right).
From Au 4f spectrum, it can be see that gold exists in two forms: Au (0) and Au (I). From C1s spectrum, we can see C-O, C-C and C-H bonds. From S2p spectrum, we can see C-S and C-S-Au bonds, as expected. From O1s spectrum, we can see C-O bonds. Although C=O bonds are also located in the band shown in the O1s spectrum, this bond cannot be seen in the C1s spectrum, therefore, we conclude that C=O is not present on the surface of this gold nanoparticles (as expected) instead a Nitrogen band can be recognized coming from the thiol. In the Cl spectrum we can recognize bands associated with KCl, which was present in the nanoparticle dispersion and must have precipitated during the drying of the sample.

Figure S13. XPS spectra of Au20PEG: C1s (top left); Au4f (top right); S2p (bottom left), O1s (bottom right).
Gold also exists in two forms: Au(0) and Au(I), as shown in the Au4f spectrum. In the S2p spectrum, we can see weak bands that can be assigned to C-S and C-S-Au bonds by which PEG and Au nanoparticles are linked. In the spectra of C1s and O1s, we can see the peaks of C-O, C-C, C-H and C=O bonds which are from PEG.

Figure S14. XPS spectra of B-1) CuO-Core,B-2) CuO^{NH2}, B-3) CuO^{COOH}, B-4) CuO^{PEG} Cu2p (top left), O1s (top right), C1s (bottom left) and N1s (bottom right).
In the spectra are shown the expected positions of the Cu(0), Cu(I) and Cu(II) assigned at around 932.6 eV, 932.4 eV and 933.6 eV, respectively. The shape of the shake-up peaks suggest that CuO-Core (sample B-1) is a mix Cu2O+Cu (in this case, the absence of shake-up peak); CuO-Ammonium and CuO-PEG (Sample B2 and B3) are a mix of CuO+ Cu2O; and CuO-Carboxylate is Cu(OH)2) (sample B-4).
In the C1s spectra we can see C-C, C-O and C=O bonds. the expected positions of the C C and C H sp3 and sp2 bonds are at around 285.0 eV, C OH bonds at around 286.5 eV and C=O and O=C OH

bonds at around 288.5 eV. Larger intensities are recorded for the CuO^{PEG} as it could be expected since they have the larger Carbon amount in the PEG chains. For the carboxylate modified CuO the C 1s band is located in the region corresponding to C in acid groups as it could also be expected.
In the O1s spectra of the samples, we can see that all samples have Cu-O, C=O and C-O bonds with different proportions. The expected positions of the Cu-O at around 529.8 eV and C=O and C O at around 531.2 eV and 532.6 eV, are also shown. For CuO^{COOH} and CuO^{PEG} only bands corresponding to C-O bounds can be detected. The CuO bands are clearly seen for the unmodified CuO-core.

Figure S15. XPS spectra of ND^{COOH}, ND^{NH2}, and ND^{PEG}: C1s (top left), N1s (top right), and O1s (bottom left).
In the C1s spectra, one peak corresponding to the C-C and C-H bonds appeared in all three samples (ND^{COOH}, ND^{NH2}, and ND^{PEG}). No significant difference can be found among samples. In the spectra of N1s, we can see C-N bonds for all the samples, but the intensity of C-N bond for $ND^{NH2}$is much stronger than the ones for the other two nanodiamonds, as a result of the presence of amine groups on its surface. A peak corresponding ti NH3+ was found for the other two samples but less-resolved. In the O1s spectra, the band located at around 531eV can be assigned to the C=O and C-O. This peak is stronger for NDPEG because there is one C-O bond per each monomer of PEG.

Figure S1.

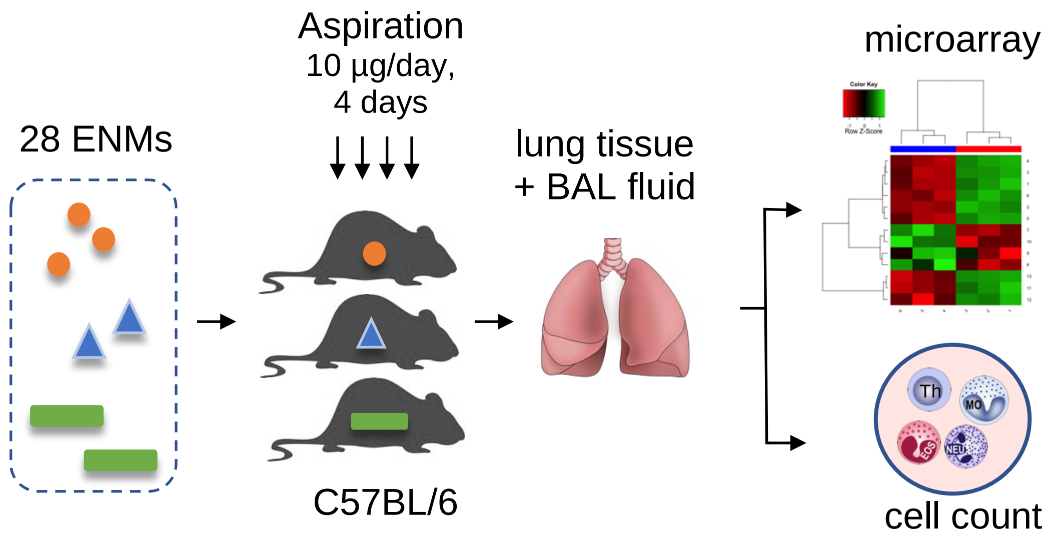
Aspiration
10 µg/day,
4 days
28 ENMs
lung tissue
+ BAL fluid
microarray
Th
MO
EOS
NEU
C57BL/6
cell count

Figure S2.

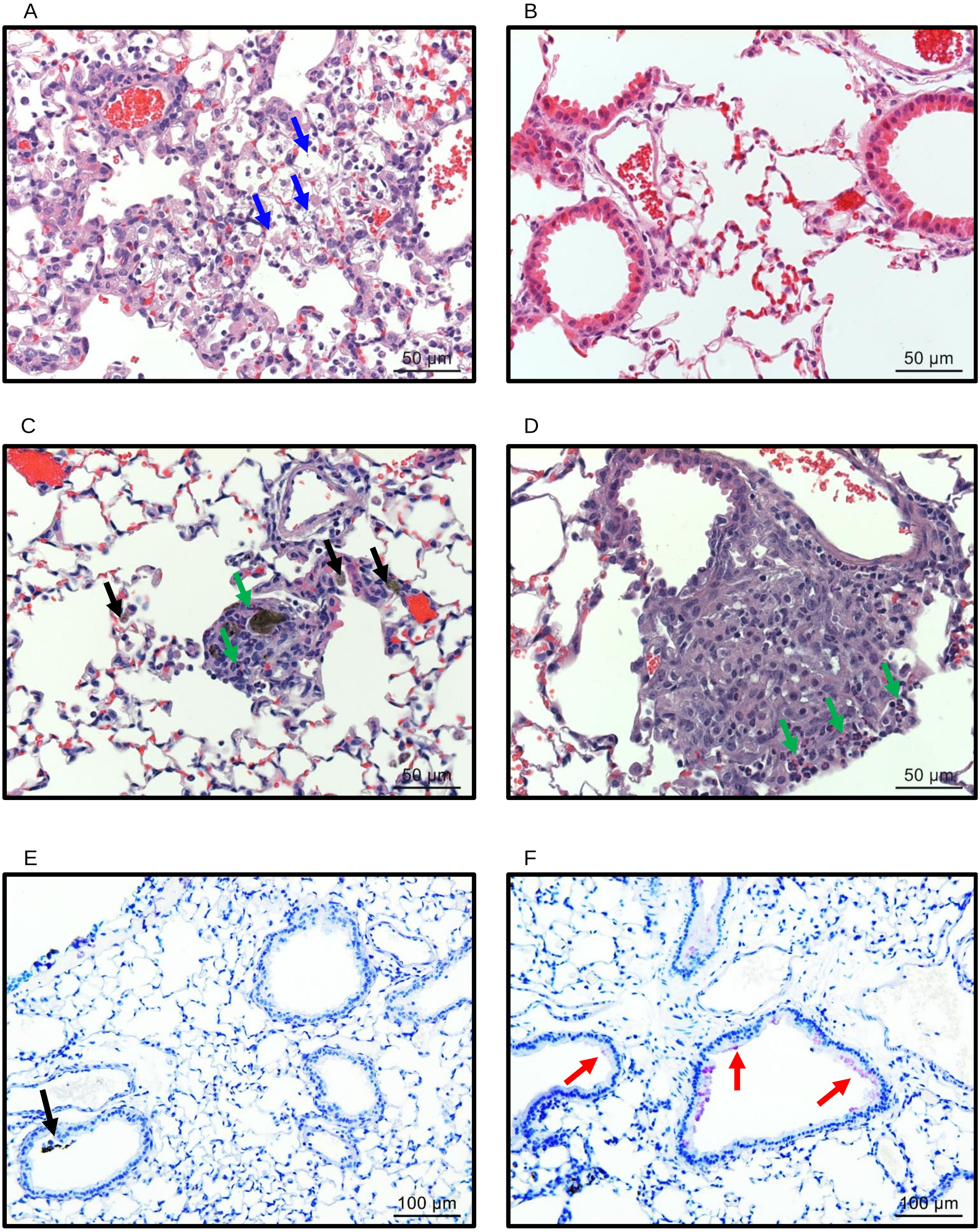

Figure S3.

A

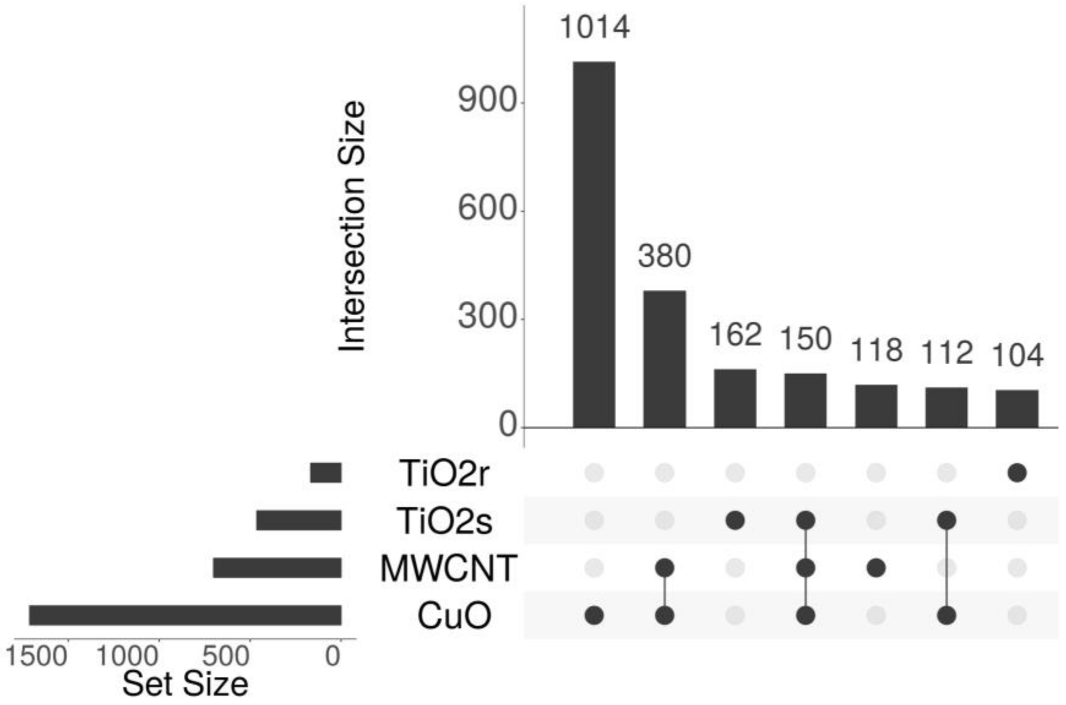
1014
380
162
150
118
112
104
Intersection Size
900
600
300
0
TiO2r
TiO2s
MWCNT
CuO
1500
1000
500
0
Set Size

B

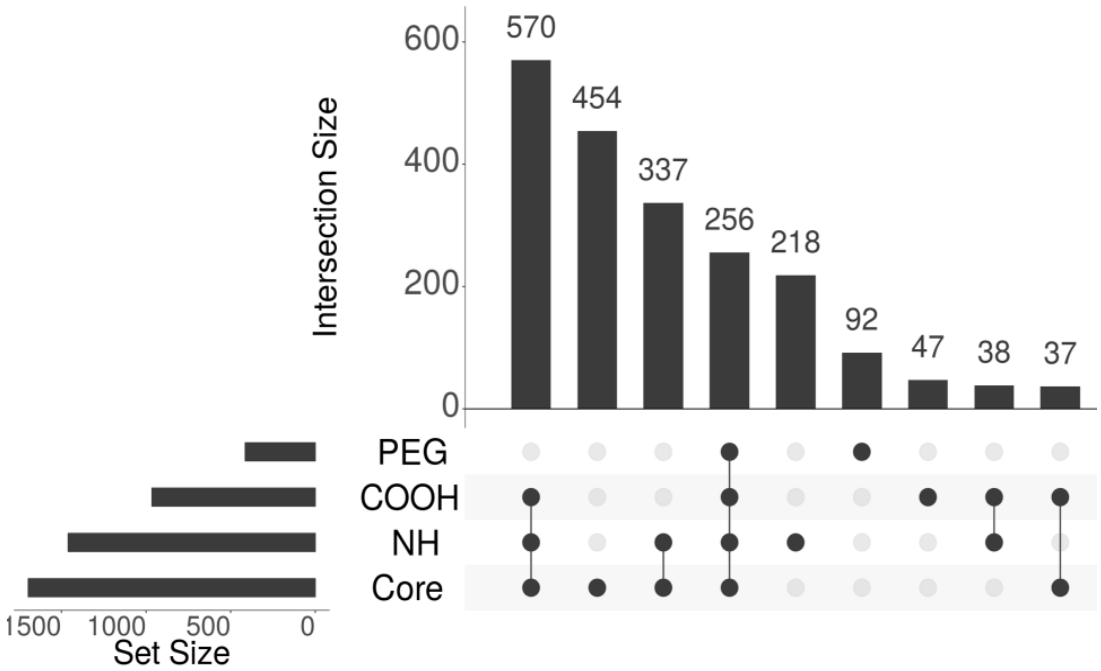
570
454
337
256
218
92
47
38
37
Intersection Size
600
400
200
0
PEG
COOH
NH
Core
1500
1000
500
0
Set Size

Figure S4.

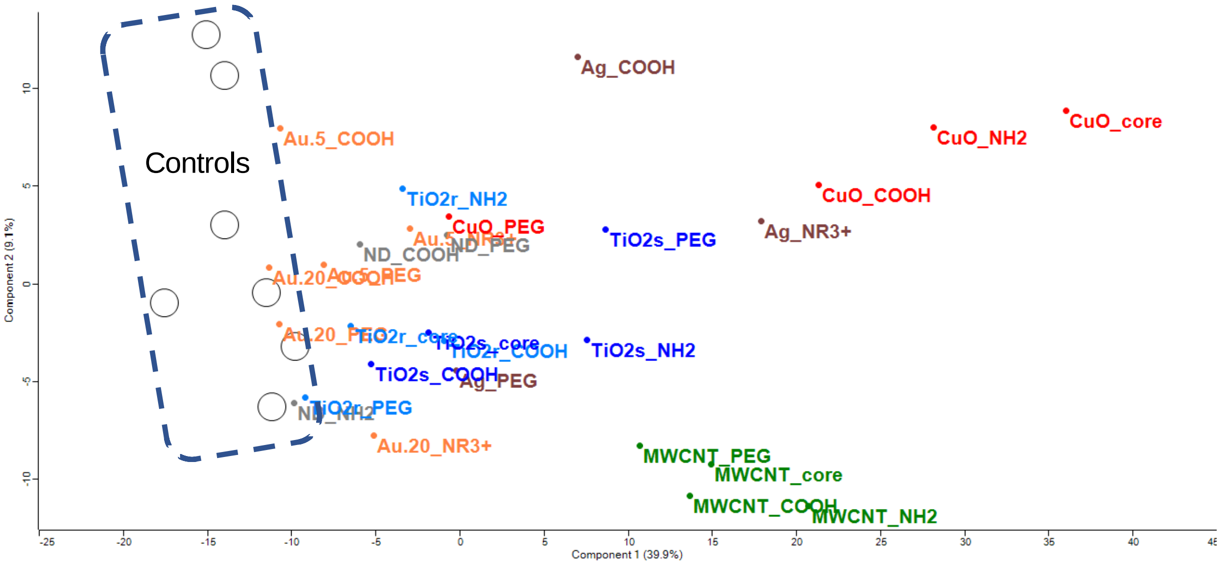
Controls
Ag_COOH
CuO_core
CuO_NH2
Au.5_COOH
CuO_COOH
TiO2r_NH2
CuO_PEG
TiO2s_PEG
Ag_NR3+
ND_COOH
TiO2s_NH2
TiO2s_COOH
Ag_PEG
Au.20_NR3+
MWCNT_PEG
MWCNT_core
MWCNT_COOH
MWCNT_NH2
Component 1 (39.9%)
Component 2 (9.1%)

Figure S5.

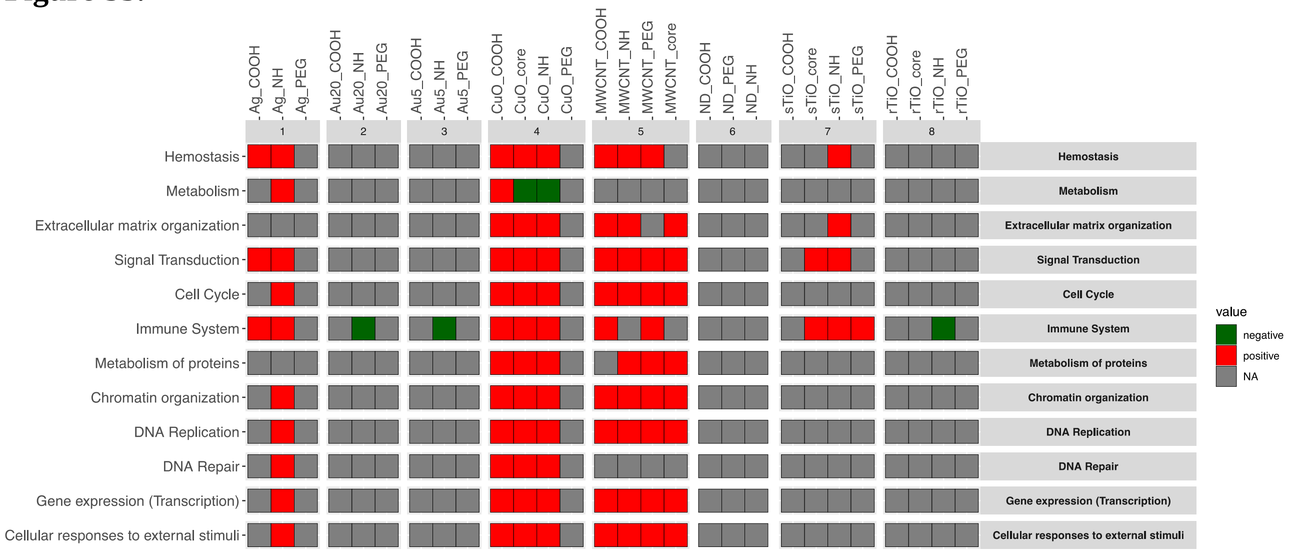
Ag_COOH
Ag_NH
Ag_PEG
Au20_COOH
Au20_NH
Au20_PEG
Au5_COOH
Au5_NH
Au5_PEG
CuO_COOH
CuO_core
CuO_NH
CuO_PEG
MWCNT_COOH
MWCNT_NH
MWCNT_PEG
MWCNT_core
ND_COOH
ND_PEG
ND_NH
sTiO_COOH
sTiO_core
sTiO_NH
sTiO_PEG
rTiO_COOH
rTiO_core
rTiO_NH
rTiO_PEG
1
2
3
4
5
6
7
8
Hemostasis
Metabolism
Extracellular matrix organization
Signal Transduction
Cell Cycle
Immune System
Metabolism of proteins
Chromatin organization
DNA Replication
DNA Repair
Gene expression (Transcription)
Cellular responses to external stimuli
value
negative
positive
NA

Figure S6.

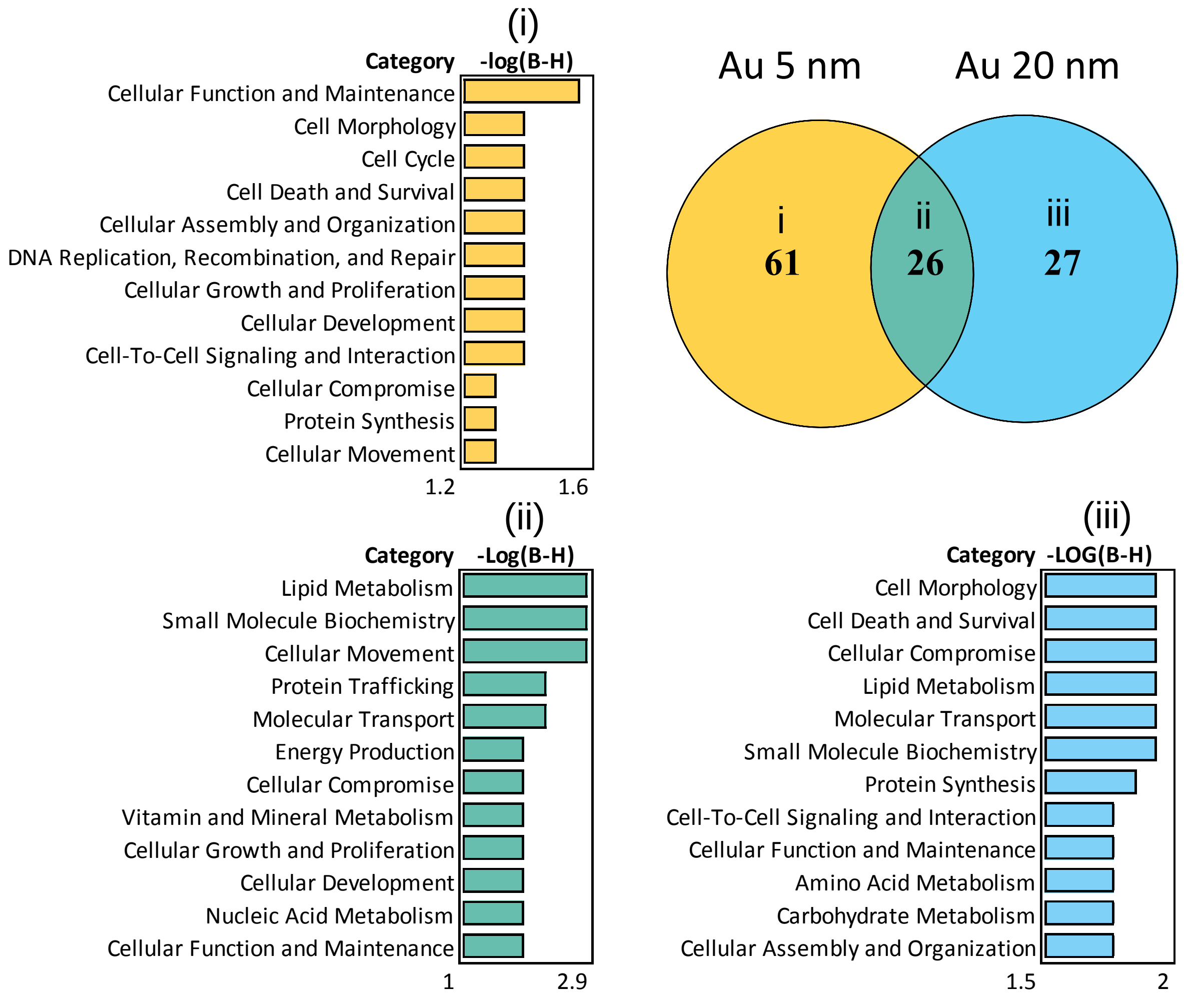
(i)
Category
-log(B-H)
Cellular Function and Maintenance
Cell Morphology
Cell Cycle
Cell Death and Survival
Cellular Assembly and Organization
DNA Replication, Recombination, and Repair
Cellular Growth and Proliferation
Cellular Development
Cell-To-Cell Signaling and Interaction
Cellular Compromise
Protein Synthesis
Cellular Movement
1.2
1.6
Au 5 nm
Au 20 nm
i
61
ii
26
iii
27
(ii)
Category
-Log(B-H)
Lipid Metabolism
Small Molecule Biochemistry
Cellular Movement
Protein Trafficking
Molecular Transport
Energy Production
Cellular Compromise
Vitamin and Mineral Metabolism
Cellular Growth and Proliferation
Cellular Development
Nucleic Acid Metabolism
Cellular Function and Maintenance
1
2.9
(iii)
Category
-LOG(B-H)
Cell Morphology
Cell Death and Survival
Cellular Compromise
Lipid Metabolism
Molecular Transport
Small Molecule Biochemistry
Protein Synthesis
Cell-To-Cell Signaling and Interaction
Cellular Function and Maintenance
Amino Acid Metabolism
Carbohydrate Metabolism
Cellular Assembly and Organization
1.5
2

Figure S7.

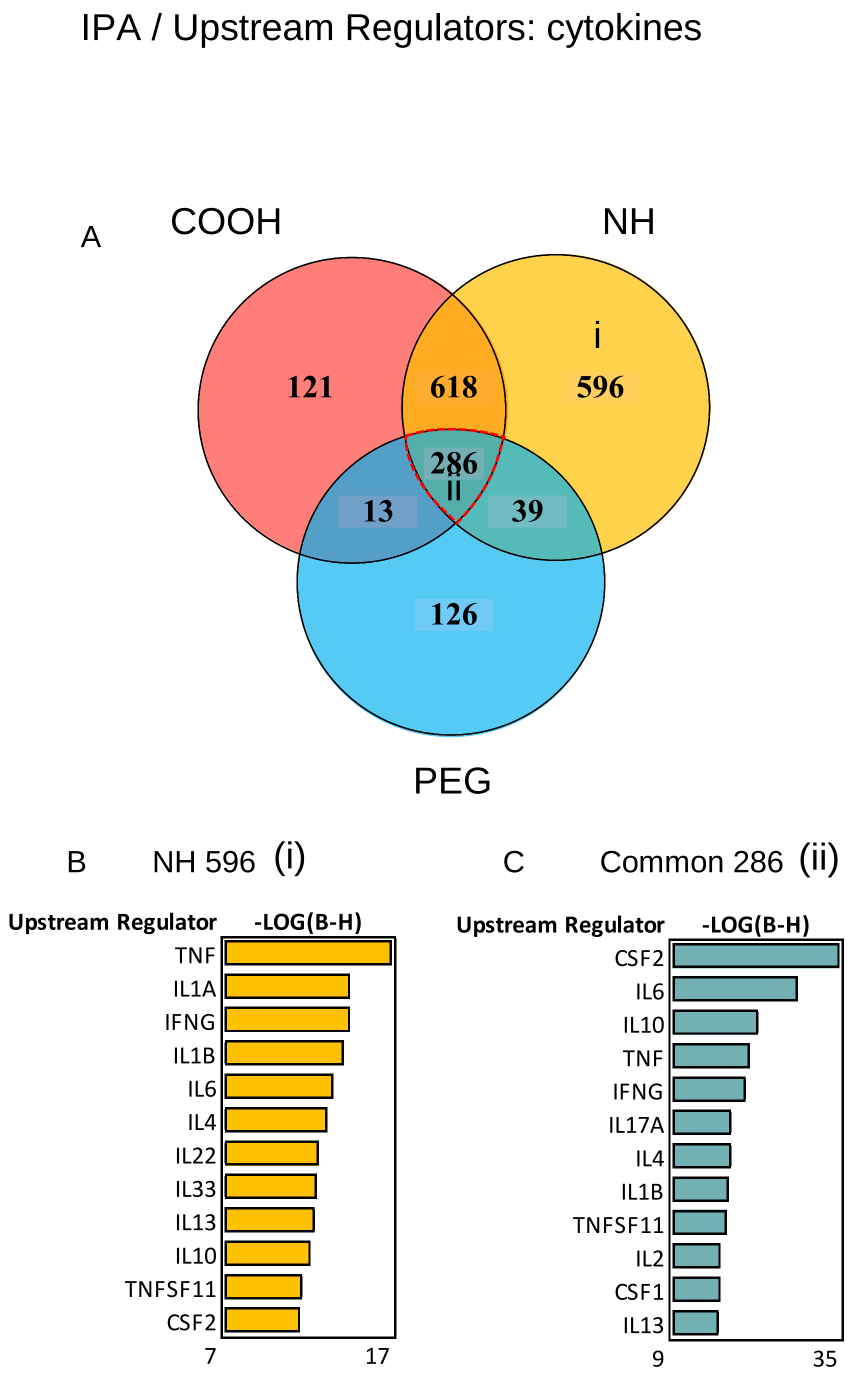
IPA / Upstream Regulators: cytokines
A
COOH
NH
PEG
121
618
596
i
286
ii
13
39
126
B
NH 596 (i)
Upstream Regulator
-LOG(B-H)
TNF
IL1A
IFNG
IL1B
IL6
IL4
IL22
IL33
IL13
IL10
TNFSF11
CSF2
7
17
C
Common 286 (ii)
Upstream Regulator
-LOG(B-H)
CSF2
IL6
IL10
TNF
IFNG
IL17A
IL4
IL1B
TNFSF11
IL2
CSF1
IL13
9
35

Figure S8.

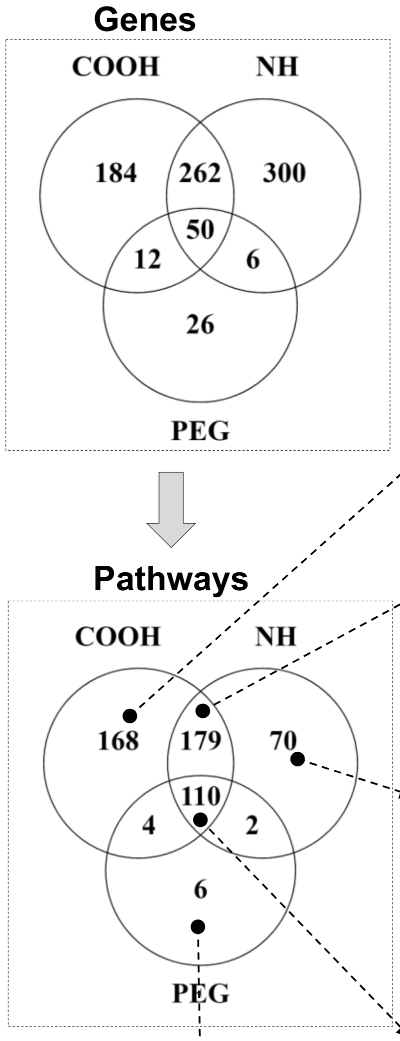

GO biological process	COOH
leukocyte chemotaxis (GO:0030595)	17.9
leukocyte migration (GO:0050900)	12.9
granulocyte chemotaxis (GO:0071621)	9.9
myeloid leukocyte migration (GO:0097529)	9.5
neutrophil chemotaxis (GO:0030593)	9.0

GO biological process	COOH	NH
response to stress (GO:0006950)	28.9	24.6
DNA replication (GO:0006260)	25.0	37.0
protein-DNA complex assembly (GO:0065004)	19.1	21.0
inflammatory response (GO:0006954)	18.5	13.9
cellular response to DNA damage stimulus (GO:0006974)	18.0	20.5

GO biological process	NH
regulation of DNA-dependent DNA replication (GO:0090329)	12.2
DNA unwinding involved in DNA replication (GO:0006268)	7.2
DNA-dependent DNA replication maintenance of fidelity (GO:0045005)	6.4
glucosamine-containing compound metabolic process (GO:1901071)	6.4
organonitrogen compound biosynthetic process (GO:1901566)	6.1

GO biological process	COOH	NH	PEG
cell cycle (GO:0007049)	86.1	65.6	59.7
mitotic cell cycle (GO:0000278)	79.5	65.8	68.2
nuclear chromosome segregation (GO:0098813)	52.0	35.6	34.4
sister chromatid segregation (GO:0000819)	45.0	31.2	38.6
mitotic sister chromatid segregation (GO:0000070)	44.2	30.5	35.3
chromosome organization (GO:0051276)	38.4	38.9	20.0
regulation of chromosome segregation (GO:0051983)	33.4	23.2	9.2
regulation of cell cycle (GO:0051726)	27.3	21.3	22.6
DNA conformation change (GO:0071103)	24.7	15.9	6.7

GO biological process	PEG
regul. of protein kinase activity (GO:0045859)	8.7
regul. of protein serine/threonine kinase activity (GO:0071900)	6.1
neg. regul. of DNA-binding transcription factor activity (GO:0043433)	4.8
regul.of microtubule cytoskeleton organization (GO:0070507)	3.6
pos. regul. of cyclin-dep. protein serine/threonine kinase activity (GO:0045737)	3.3

Figure S9 XPS spectras TiO_2s^{COOH}

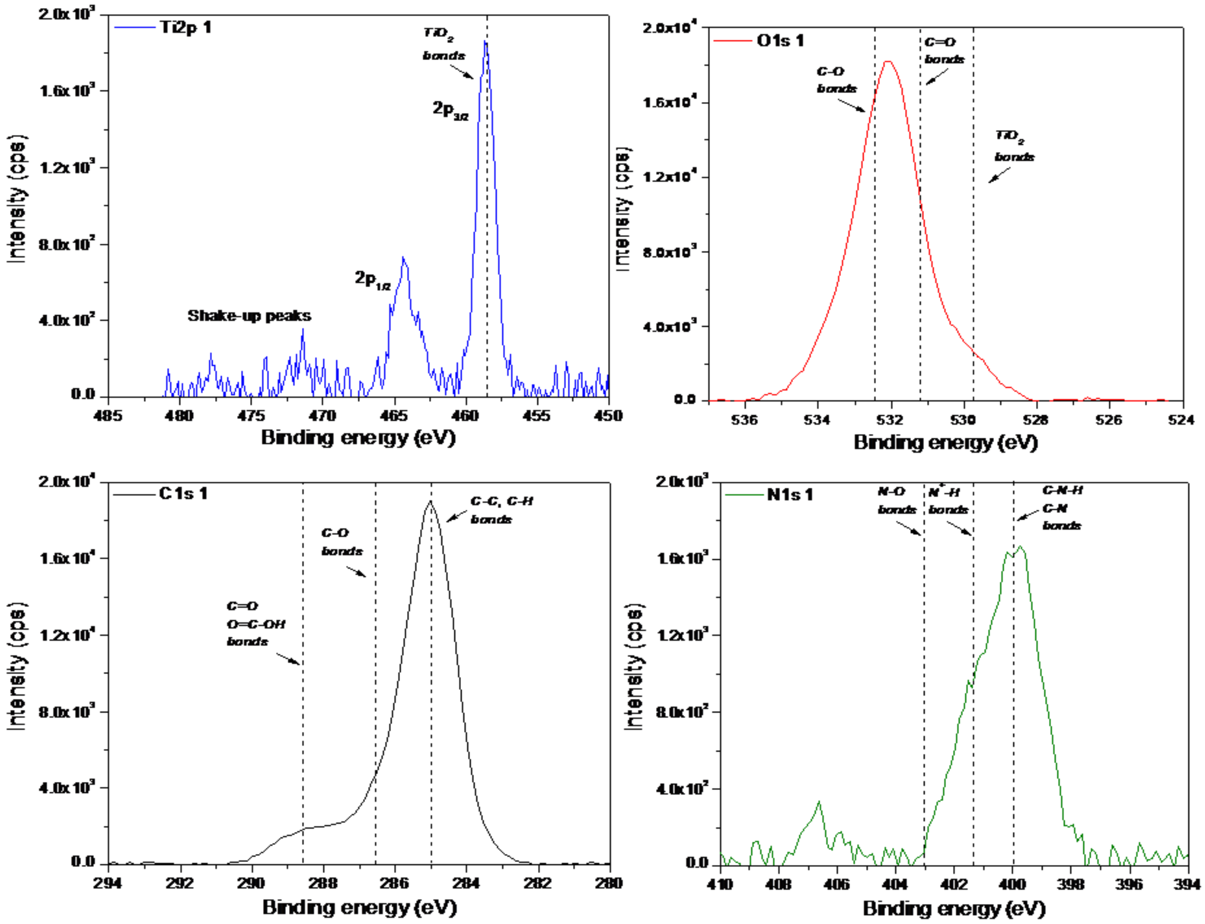

Figure S9 XPS spectra of TiO_2s^{COOH} : Ti2p (top left); O1s (top right); C1s (bottom left) and N1s (bottom right).

From Ti2p and O1s spectra, it can be seen that the ENM are mainly composed of TiO_2. From C1s and O1s spectra, we can see the presence of C-H and C-C bonds, as well as C-O and C=O-OH bonds, indicating the existence of –COOH groups on the nanoparticles surface. We can also see the –N-H and C-N-H bonds in the N1s spectrum, which is also supposed to appear according to the functionalization process of the nanoparticles. ((TiO_2)-CH_2-CH_2-$CH_2NHOC(O)CH_2CH_2COOH$)

Figure S10 XPS spectras TiO_2s^{NH2}

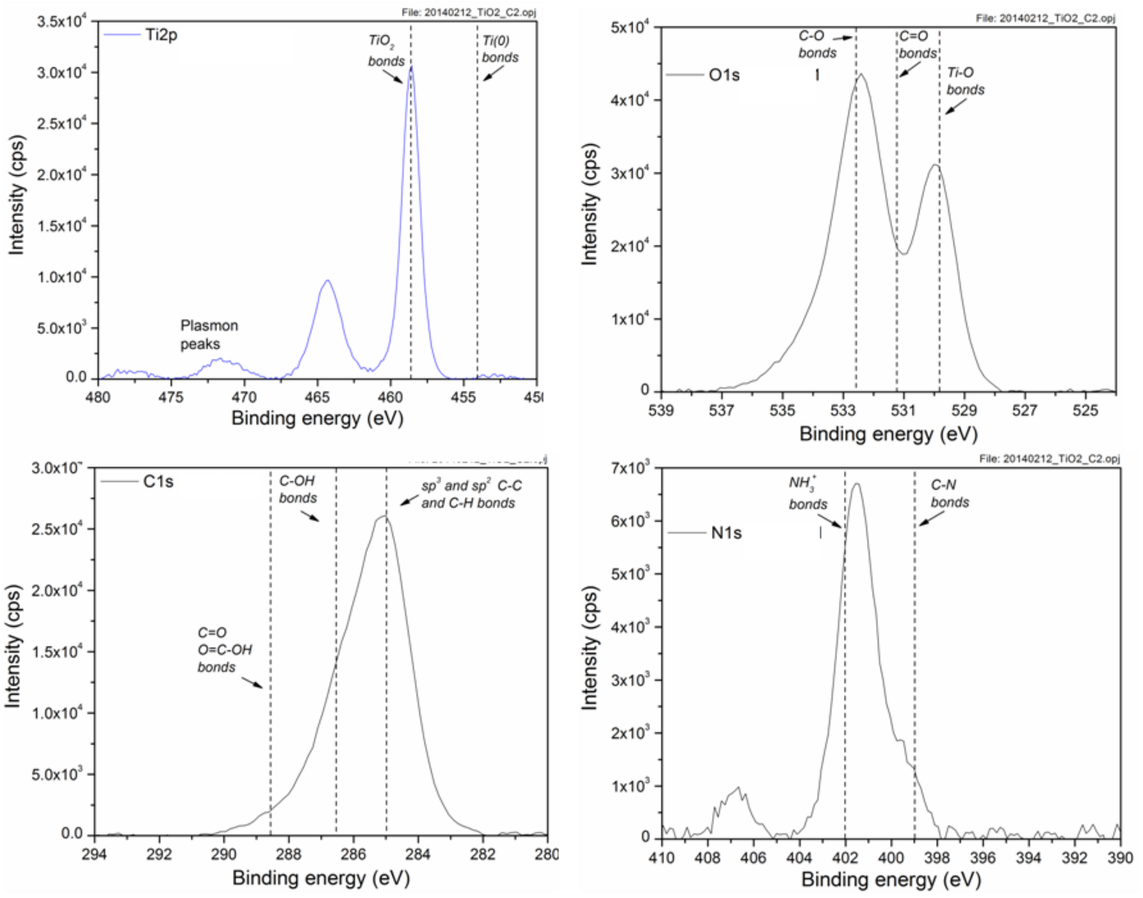

Figure 110 - XPS spectra of TiO_2s^{NH2} Ti2p (top left); O1s (top right); C1s (bottom left) and N1s (bottom right).

For TiO_2s^{NH2} their surface chemistry is supposed to be (TiO_2)-Si-$CH_2CH_2CH_2NH_2$ after the surface modification. As shown in the Ti2p and O1s spectra above, Ti-O bonds in Ti 2p and O1s spectra show the nanoparticles are TiO_2. In C1s spectrum, the band located at 285eV can be assigned to the sp3 and sp2 C-C and C-H bonds, from the N1s spectrum, C-N bonds and NH3+ can also be found in the band ranging from 398-404eV. These results confirmed that NH3+ is present on the surface of the TiO_2s^{NH2} nanoparticles.

Figure S11 XPS spectras Au20^{COOH}

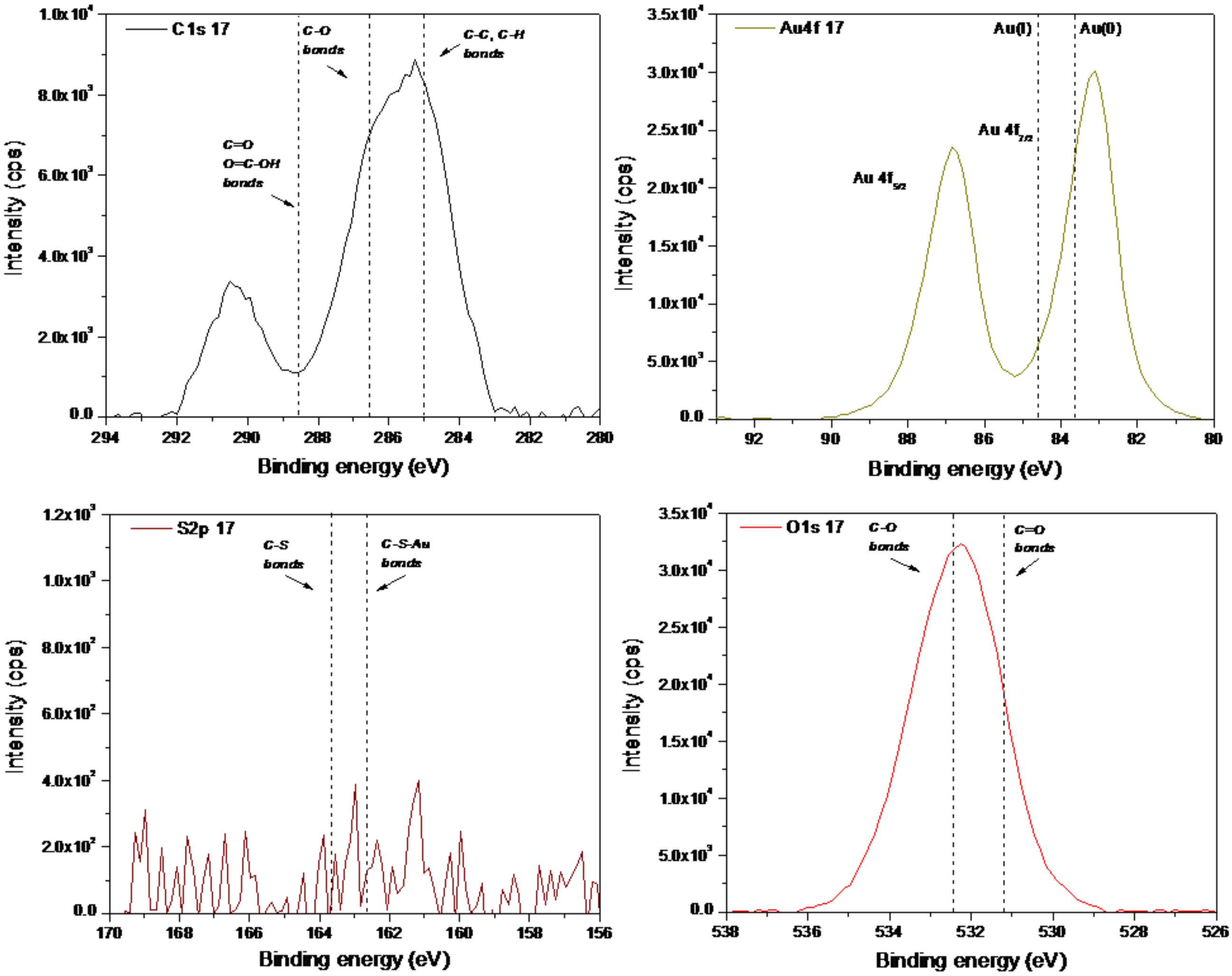

Figure S11 - XPS spectra of Au20^{COOH}: C1s (top left); Au4f (top right); S2p (bottom left), O1s (bottom right).

From the Au4f spectrum, we can see that gold exists mostly in the form of Au(0) and there are possibly a small amount of Au (I) in this sample. We observe a strong peak for C=O bonds and we can also see C-O (O1s spectrum), C-C and C-H (C1s spectrum).

Figure S12 XPS spectras Au20^{NR3}

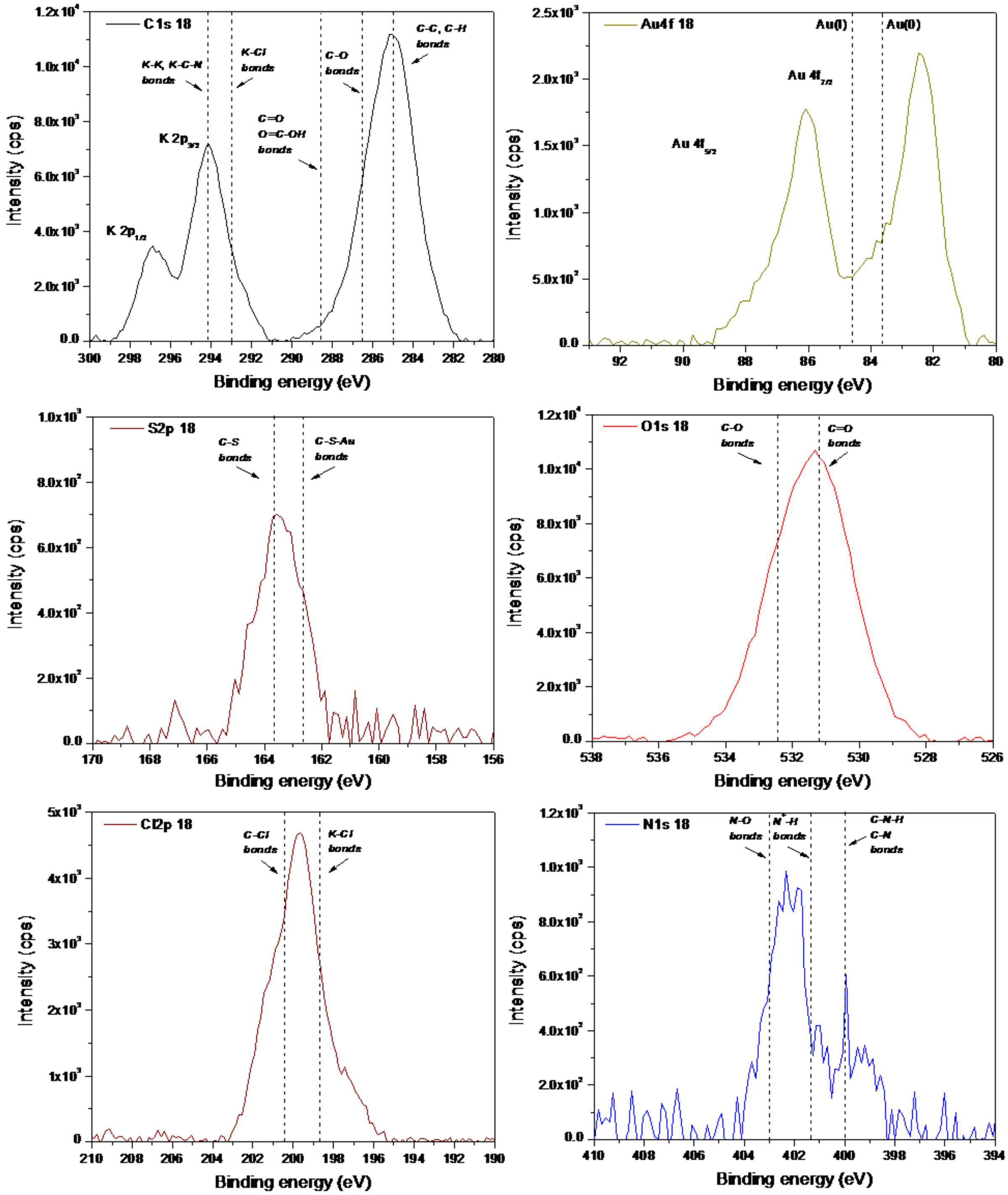

Figure S12 2 - XPS spectra of Au20^{NR3}C1s (top left); Au4f (top right); S2p (middle left), O1s (middle right), Cl2p (bottom left) and N1s (bottom right).

From Au 4f spectrum, it can be see that gold exists in two forms: Au (0) and Au (I). From C1s spectrum, we can see C-O, C-C and C-H bonds. From S2p spectrum, we can see C-S and C-S-Au bonds, as expected. From O1s spectrum, we can see C-O bonds. Although C=O bonds are also located in the band shown in the O1s spectrum, this bond cannot be seen in the C1s spectrum, therefore, we conclude that C=O is not present on the surface of this gold nanoparticles (as expected) instead a Nitrogen band can be recognized coming from the thiol. In the Cl spectrum we can recognize bands associated with KCl, which was present in the nanoparticle dispersion and must have precipitated during the drying of the sample.

Figure S13 XPS spectras Au20PEG

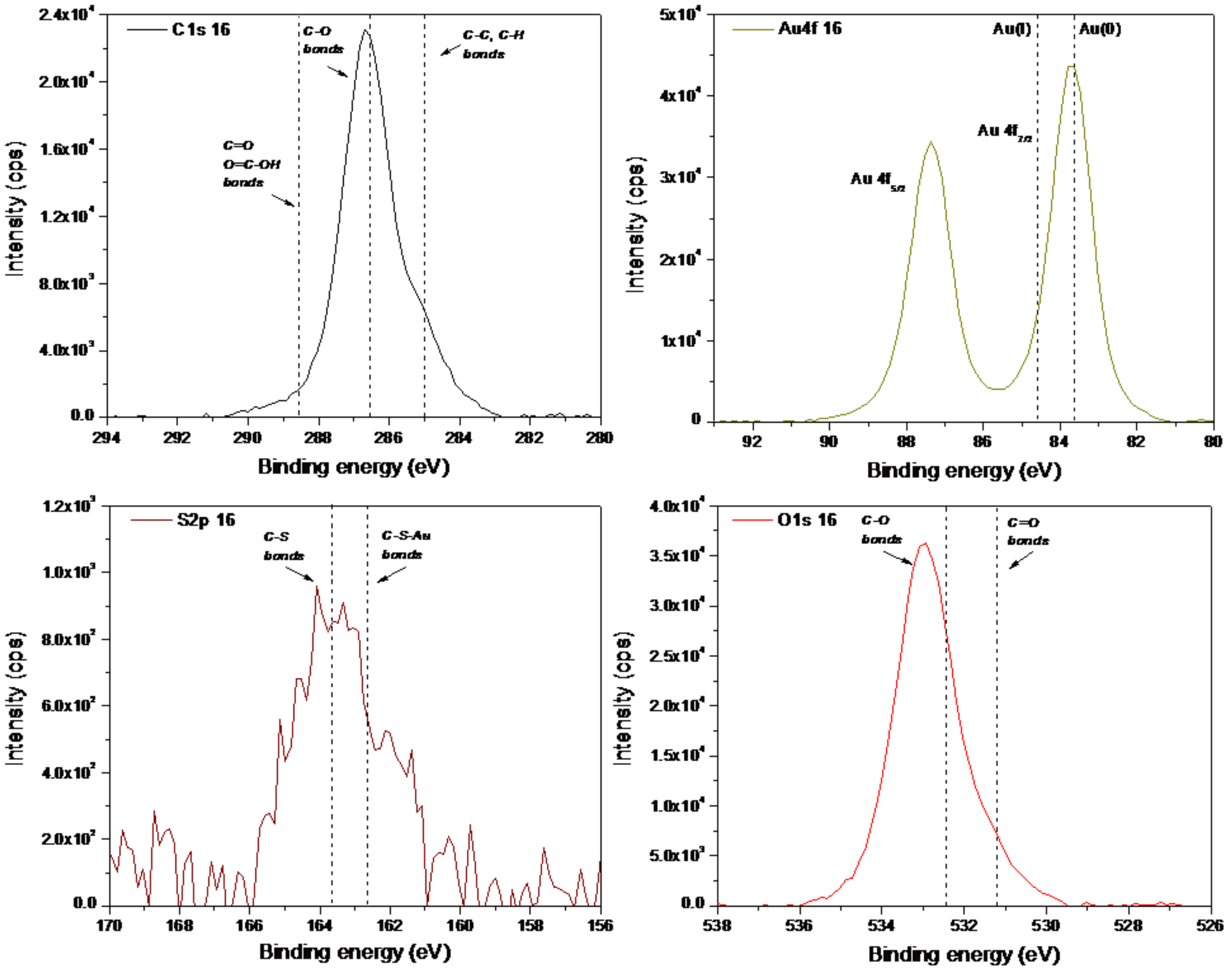

Figure S13 - XPS spectra of Au20$^{PEG:}$ C1s (top left); Au4f (top right); S2p (bottom left), O1s (bottom right).

Gold also exists in two forms: Au(0) and Au(I), as shown in the Au4f spectrum. In the S2p spectrum, we can see weak bands that can be assigned to C-S and C-S-Au bonds by which PEG and Au nanoparticles are linked. In the spectra of C1s and O1s, we can see the peaks of C-O, C-C, C-H and C=O bonds which are from PEG.

Figure S14 XPS spectras CuO

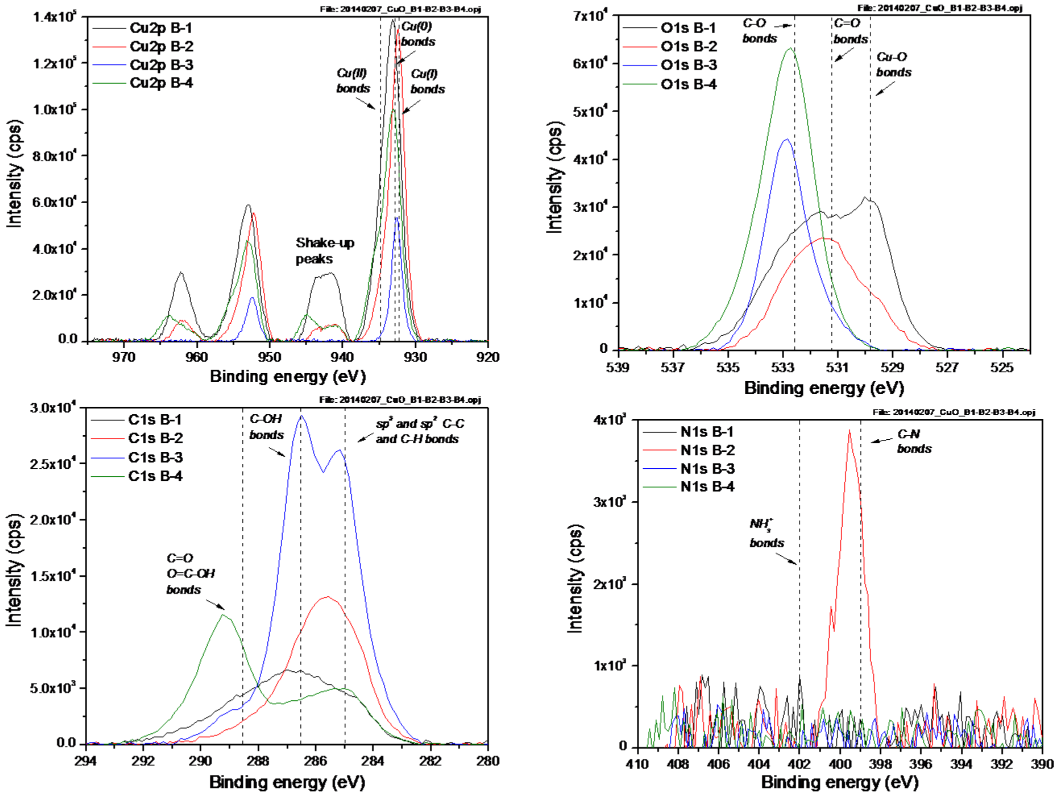

Figure S14 - XPS spectra of B-1) CuO-Core,B-2) CuO^{NH2}, B-3) CuO^{COOH}, B-4) CuO^{PEG} Cu2p (top left), O1s (top right), C1s (bottom left) and N1s (bottom right).

In the spectra are shown the expected positions of the Cu(0), Cu(I) and Cu(II) assigned at around 932.6 eV, 932.4 eV and 933.6 eV, respectively. The shape of the shake-up peaks suggest that CuO-Core (sample B-1) is a mix Cu_2O+Cu (in this case, the absence of shake-up peak); CuO-Ammonium and CuO-PEG (Sample B2 and B3) are a mix of CuO+ Cu2O; and CuO-Carboxylate is $Cu(OH)_2$) (sample B-4).

In the C1s spectra we can see C-C, C-O and C=O bonds. the expected positions of the C-C and C-H sp3 and sp2 bonds are at around 285.0 eV, C-OH bonds at around 286.5 eV and C=O and O=C-OH bonds at around 288.5 eV. Larger intensities are recorded for the CuO^{PEG} as it could be expected since they have the larger Carbon amount in the PEG chains. For the carboxylate modified CuO the C 1s band is located in the region corresponding to C in acid groups as it could also be expected.

In the O1s spectra of the samples, we can see that all samples have Cu-O, C=O and C-O bonds with different proportions. The expected positions of the Cu-O at around 529.8 eV and C=O and C-O at around 531.2 eV and 532.6 eV, are also shown. For CuO^{COOH} and CuO^{PEG} only bands corresponding to C-O bounds can be detected. The CuO bands are clearly seen for the unmodified CuO-core.

Figure S15 XPS spectras Nanodiamonds

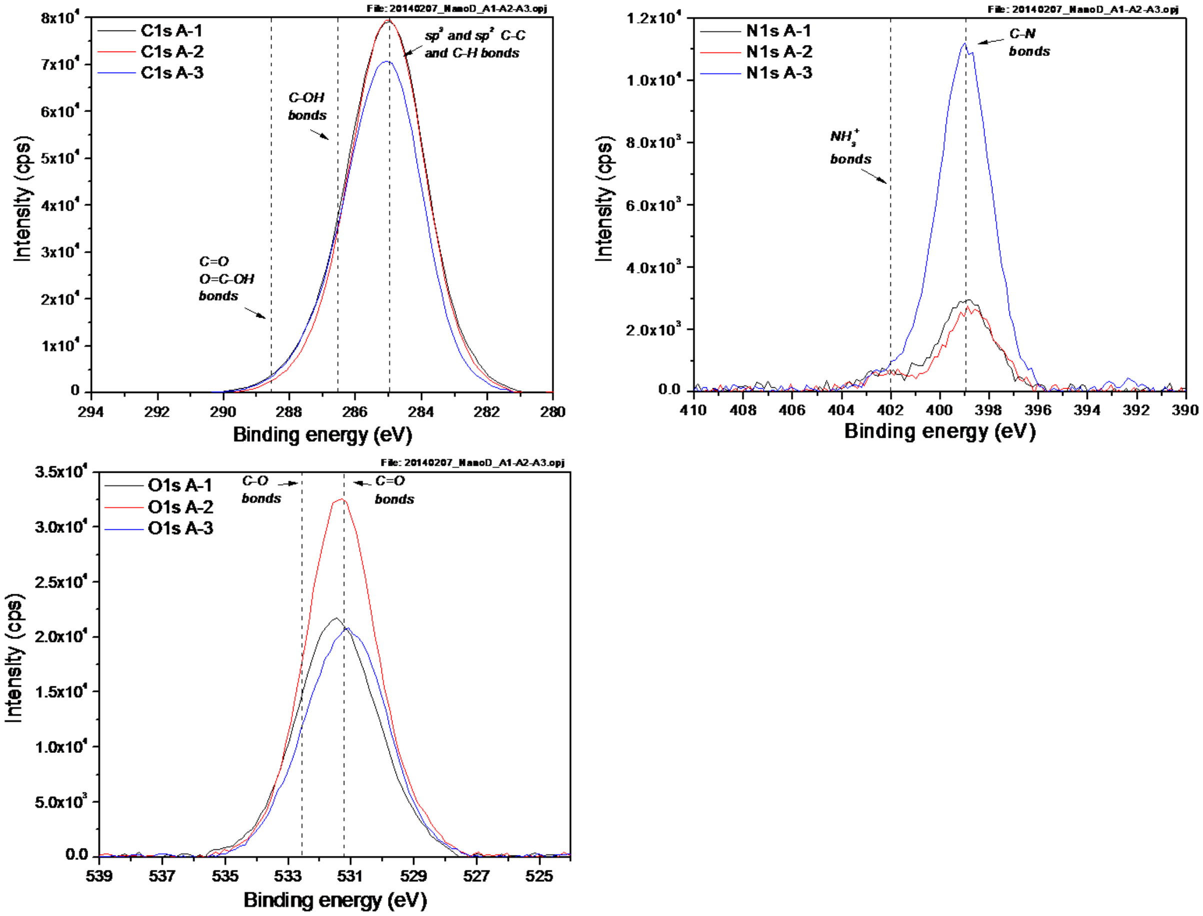

Figure S15 3 - XPS spectra of ND^{COOH}, ND^{NH2}, and ND^{PEG}: C1s (top left), N1s (top right), and O1s (bottom left).

In the C1s spectra, one peak corresponding to the C-C and C-H bonds appeared in all three samples (ND^{COOH}, ND^{NH2}, and ND^{PEG}). No significant difference can be found among samples. In the spectra of N1s, we can see C-N bonds for all the samples, but the intensity of C-N bond for $ND^{NH2}$is much stronger than the ones for the other two nanodiamonds, as a result of the presence of amine groups on its surface. A peak corresponding ti NH_3^+ was found for the other two samples but less-resolved. In the O1s spectra, the band located at around 531eV can be assigned to the C=O and C-O. This peak is stronger for ND^{PEG} because there is one C-O bond per each monomer of PEG.

Table S1. Summary of XPS characterization of ENMs

ENM	Presence of Carboxyl groups	Presence of amine groups	Presence of PEG
MWCNT^{Core}	No	No	No
MWCNT^{COOH}	Significant CO related bands indicate presence of COOH		
MWCNT^{NH2}		Not detected	
MWCNT^{PEG}			Significant CO related bands indicate presence of PEG
CuO^{Core}	No	No	No
CuO^{COOH}	CO related bands indicate presence of COOH		
CuO^{NH2}		Characteristic amine bands	
CuO^{PEG}			High intensity of CO bands
TiO_2s^{Core}	No	No	No
TiO_2s^{NH2}		Characteristic amine bands	
TiO_2s^{PEG}			High intensity of CO bands
TiO_2s^{COOH}	CO related bands indicate presence of COOH		
TiO_2r^{Cor}	No	No	No
TiO_2r^{COOH}	Intense CO related bands		
TiO_2r^{NH2}		Weak N bands	
TiO_2r^{PEG}			High intensity of CO bands
Au20^{NR3}		N bands	
Au20^{PEG}			High intensity of CO bands
Au20^{COOH}	CO related bands indicate presence of COOH		
ND^{NH2}		No specific bands detected	
ND^{COOH}	No specific bands detected		
ND^{PEG}			High intensity of CO bands
